# Erk1^R84H^ is an oncoprotein that causes hepatocellular carcinoma in mice and imposes a rigorous negative feedback loop

**DOI:** 10.1038/s41388-025-03437-6

**Published:** 2025-05-20

**Authors:** Nadine Soudah, Alexey Baskin, Merav Darash-Yahana, Ilona Darlyuk-Saadon, Karina Smorodinsky-Atias, Tali Shalit, Wei-ping Yu, Alon Savidor, Eli Pikarsky, David Engelberg

**Affiliations:** 1 Department of Biological Chemistry, Alexander Silberman Institute of Life Sciences, The Hebrew University of Jerusalem, Jerusalem, Israel; 2 CREATE-NUS-HUJ Mechanisms of Liver Inflammatory Diseases, National University of Singapore, 1 CREATE WAY, Innovation Wing, Singapore, Singapore; 3 Department of Microbiology, Yong loo lin School of Medicine, National University of Singapore, Singapore, Singapore; 4 School of Neurobiology, Biochemistry and Biophysics, Tel Aviv University, Tel Aviv-Yafo, 6997801 Israel; 5 Nancy and Stephen Grand Israel National Center for Personalized Medicine, Weizmann Institute of Science, Rehovot, 7610001 Israel; 6 Animal Gene Editing Laboratory (AGEL), Biological Resource Centre, Agency for Science, Technology and Research (A*STAR), Proteos, 138673 Singapore, Singapore; 7 Institute of Molecular and Cell Biology, Agency for Science, Technology and Research (A*STAR), Proteos, 138673 Singapore, Singapore; 8 Department of Immunology and Cancer Research and Department of Pathology, Institute for Medical Research Israel-Canada, Hadassah Medical School - Hebrew University, Jerusalem, Israel

**Keywords:** Extracellular signalling molecules, Cancer models

## Abstract

The receptor tyrosine kinase (RTK)-Ras-Raf-MEK-Erk cascade is frequently mutated in cancer, but it is not known whether Erk is a sole mediator of the pathway’s oncogenicity, and what degree of Erk activity is required for oncogenicity. Also, it is assumed that high Erk activity is required to impose and maintain oncogenicity, but the exact degree of required activity is not clear. We report that induced expression of the intrinsically active variant Erk1^R84H^ in mouse liver gave rise to hepatocellular carcinoma (HCC). Intriguingly, the phosphorylated/active form of Erk1^R84H^ was dramatically downregulated during HCC development, and became almost undetectable in mature tumors. Similarly, in Erk1^R84H^-transformed NIH3T3 cells, the phosphorylated/active form of Erk1^R84H^ was undetectable. Thus, 1) Erk1 could by itself cause HCC in mice, suggesting that it is the major or even the sole mediator of the cascade’s oncogenicity. 2) Erk1^R84H^-induced tumors (and other tumors) are maintained by a minimal Erk activity. 3) Erk1^R84H^ is probably the driver of the malignancy in patients that carry the R84H mutation.

## Introduction

The extracellular signal-regulated kinases (Erks), Erk1 and Erk2, are the downstream targets of the proto-oncogenic cascade, known as the receptor tyrosine kinase (RTK)-Ras-Raf-MEK pathway. All components of the cascade were found mutated and uncontrollably active in various cancers [1–6]. The mutated proteins were shown to oncogenically transform cells in culture and to give rise to tumors in transgenic mice [7–14], strongly suggesting that they play a causative role in the human maladies.

Assuming linearity of the pathway would imply that levels of activity of upstream components (e.g., RTK, Ras, Raf) are proportionally translated to the level of Erk activity, and to the severity of the oncogenic outcome. The notion of pathway linearity further implies that Erks are the major, if not the only, mediators of the pathway oncogenicity. This presumption is supported by observations of high Erk activity in many cancer cases and models [15–21].

If Erks are indeed relaying the pathway’s oncogenicity, then activation of Erk itself, without activation of any upstream component, should give rise to tumors. This, however has not been shown.

On the basis of the assumption that elevated Erk activity promotes cancer, Erk inhibitors are being developed. They are believed to be efficient in blocking the pathway wherever the oncogenic mutation may occur. BVD-523 (ulixertinib), ASN-007 and LY-3214996 are such inhibitors that have reached clinical trials for the treatment of cancers with activating mutations in KRAS, BRAF, NRAS, MEK1/2, and ERK1/2 [22–27]. They have been shown to be effective in cancer cells and tumors with acquired resistance to BRAF and MEK inhibitors [28–32]. Many aspects of the effects of these drugs on cells in culture, animal models and cancer patients are still not clear.

Some observations, however, challenge the idea that high Erk activity is the singular mediator of the oncogenicity of the RTK-Ras cascade. One is the fact that in its upstream part the pathway is not linear. RTKs and Ras activate many effectors in parallel to the Raf-MEK-Erk arm, some of which are protooncogenic themselves [3, 4, 33–36].

Another is the observations that in some cancer models the relationships between the degree of pathway activation, levels of Erk activity, and severity of cancer are not proportional. It has been shown, in fact, that under some conditions the Raf-MEK-Erk pathway may promote cell death, so that a certain threshold of Erk activity determines the cell fate [37–39]. Studies with samples of colorectal cancer showed that many of them are not stained for active/phosphorylated Erk even though they carry activating mutations in Ras or B- Raf [40]. These studies suggest that a given level of Erk activity, not necessarily very high, is cancer-causing [41].

The fact that activating mutations in Erk1/2 are rarely found in cancer [42] strengthens the doubts over Erk being the major or even the sole mediator of the pathway’s oncogenicity. The most abundant mutation found in Erks in cancer is E320K (COSM461148), observed in ERK2 of several dozen squamous cell carcinoma patients. Another mutation, R84H, was detected in ERK1 in a subset of patients across different types of cancer (COSV53774085 and cBioPortal). It is not known whether these mutations play a causative role in these diseases or are merely bystanders. It is consequently not known if cancers containing mutated Erks are sensitive to Erk inhibitors and how they may affect the Erk pathway and cancer progression.

The E320K mutation does not increase Erk2’s catalytic activity and does not confer on it the capability to oncogenically transform cells in culture [43]. Its role in cancer, if any, is not clear. Erk1^R84H^, on the other hand, was shown to be biochemically intrinsically active, independent of upstream activation, both as a purified recombinant molecule and when expressed in cell lines [44]. Mutations in Arg84 were, in fact, originally identified in a screen for MEK-independent Erk molecules [45]. They bypass the need of upstream activation, as the mutations render them capable of autoactivation/autophosphorylation [46]. Various mutants in R84, primarily Erk1^R84S^ and Erk1^R84H^, were shown to oncogenically transform NIH3T3 cells [44, 46] and an equivalent mutant of *Drosophila’s* Erk (Rolled^R80S^) was shown to give rise to tumors in the fly’s eye [47]. On the basis of these observations it seems plausible that the R84H mutation is the causing element of the cancer in the patients that carry it. Such a presumption would be strongly substantiated if Erk1^R84H^ is shown to give rise to tumors in the context of the whole mammal. If it does, it would also support the view that Erks (perhaps Erk1) are the major mediators of the oncogenicity of the (RTK)-Ras-Raf-MEK cascade.

The main goal of this study was, therefore, to induce expression of Erk1^R84H^ in vivo and to determine whether cancer develops. To this end we have successfully established a transgenic mouse model in which expression of Erk1^R84H^ could be induced in a tissue-specific and temporally-controlled manner. We induced Erk1^R84H^ expression in the liver and observed that it caused, within about two months, a severe hepatocellular carcinoma (HCC).

Intriguingly, the level of phosphorylated/active form of the oncogenic Erk1^R84H^ was dramatically downregulated in the course of HCC development in this mouse model, and was almost undetectable in the mature tumors. To reveal the mechanism through which the mutated Erk1 causes oncogenic transformation and downregulates its own activity during the process, we expressed it in NIH3T3 cells, verified that it oncogenically transforms them, and followed its status and its effect on the proteome and phosphoproteome. It was found that, similar to the observation in the mouse model, Erk1^R84H^’s phosphorylation is dramatically downregulated during the process of cell transformation and becomes barely detectable at the *foci* maintenance stage. We found that suppression of the Erk1^R84H^’s active form occurs via a robust feedback inhibition that, counter intuitively, is relieved by Erk inhibitors. Testing human tumor-derived cell lines, we found that Erk phosphorylation is suppressed in most of them, but could be de-suppressed by treatment with Erk inhibitors. Analyses of the proteome and of the phosphoproteome show that Erk1^R84H^ modifies regulation of cell cycle and also activates uridine phosphorylase 1, probably as a means to use ribose as an energy source. In summary, Erk1^R84H^ is shown to be an efficient oncoprotein both in vivo and in cell culture. It activates the cell cycle machinery and mechanisms to fuel various energy sources. In both cells and animals, Erk1^R84H^ activates a powerful feedback inhibition machinery that reduces its activity to below detectable levels. This phenomenon is also observed in human cancer cells. Thus, although Erk is a major driver of oncogenesis, many tumors maintain a minimal Erk activity.

## Materials and methods

### Transgenic mice

Erk1^R84H^ transgenic mice were generated at the Animal Gene Editing Laboratory (AGEL), Biological Resource Centre, Agency for Science, Technology and Research (A*STAR), Singapore. The transgene docking cassette was targeted to the Rosa26 locus in mouse embryonic stem (ES) cells (129 background) using homologous recombination techniques. The Erk1^R84H^ cassette was subsequently integrated into the docking region by Flp Recombinase-Mediated Cassette Exchange (RMCE). ES clones were selected and used to generate chimera founders through standard blastocyst injection procedures. Chimera founders were bred with C57BL/6JInv females for germline transmission. The Alb-cre driver mice (Jackson Laboratories, stock number: 003574) were purchased from The Jackson Laboratory. For inducing transgene expression dox-supplemented diet at a concentration of 625 mg·kg^−1^ (TD.01306 Rodent Diet, Envigo) was used.

### Ethics statement

all procedures related to mouse breeding and experimentation were conducted in compliance with approved protocols (IACUC protocol number NS-23-17325-5), under the oversight of the Institutional Animal Care and Use Committee (IACUC) at The Hebrew University of Jerusalem (HUJI). All of the experiments complied with all relevant ethical regulations.

### Mice genotyping

DNA was extracted from mice ear clips using REDExtract-N-Amp Tissue PCR kit (XNAT100RXN, Sigma) following the manufacturer’s protocol. The primers used to set up the PCR reactions are shown in Table 1.

**Table 1:** Primers used to set up PCR reactions for mice genotyping.

PCR product name	Forward primer	Reverse primer	PCR length (bp)
Erk1^R84H^	ATTGGGAAGACAATAGCAGGCATGC	TCAAAGAGCAGCGAGAAGCGTTCAG	292
R26-211	TTGCCTCAAGAGGGGCGTGCTGAGCCAG	AGGACAACGCCCACACACCAGGTTAGC	378
Alb-cre WT	TGCAAACATCACATGCACAC	TTGGCCCCTTACCATAACTG	351
Alb-cre mutant	GAAGCAGAAGCTTAGGAAGATGG	TTGGCCCCTTACCATAACTG	390

PCR products corresponding to Erk1^R84H^ and R26-211 were separated on a 1% agarose gel, while the Alb-cre PCR products were separated on a 3% agarose gel.

### Mice sacrifice, tissue lysis, protein extraction and quantification

Mice were euthanized via an overdose of a cocktail consisting of 0.75 mg/mL Ketamine and 0.1 mg/mL Medetomidine in saline (administered at 0.015 mL/g body weight), followed by cardiac puncture, as per the IACUC protocol NS-23-17325-5. Organs were harvested immediately and snap-frozen in liquid nitrogen for preservation.

For protein extraction, between 500 and 700 µL of lysis buffer (50 mM HEPES pH 7.5, 150 mM NaCl, 1 mM EDTA, 1% Triton X-100, 0.1% sodium deoxycholate, 0.1% SDS) supplemented with HALT™ Protease and Phosphatase Inhibitor Cocktail (100×) (#78440; Thermo Fisher Scientific) was added to the frozen tissue samples. Tissue homogenization was performed using a Bullet Blender Tissue Homogenizer (Next Advance) with the appropriate beads for each tissue type. The resulting lysates were then mixed with a 4× SDS sample buffer and boiled for 10 min to denature the proteins.

Protein concentrations were measured using the MN Protein Quantification Assay (250) Reagents Kit (740967.250; MACHEREY-NAGAL, Duren, Germany).

### Histology

Liver tissues were harvested, and the large lobe was fixed in 20 volumes of 10% Neutral Buffered Formalin (NBF) (HT501128, Sigma) for 48 h at room temperature. After fixation, the samples were trimmed, embedded in paraffin, and sectioned. Tissue sections were subsequently stained with Hematoxylin and Eosin (H&E). Kidney tissues were also harvested and underwent the same histological process. All histological processing, including embedding, sectioning, and slide preparation, was carried out by the Histology Laboratory at the Animal Facilities in Hadassah, Ein Kerem. H&E-stained slides were examined by an ACVP board-certified veterinary pathologist who was blinded to the sample identities. Images of the stained tissue sections were captured using an Olympus BX43 light microscope and a ToupCam XCAM4K8MPB, 4K HDMI industrial digital camera, with images processed using ToupView software.

### Western blot

Protein extracts (15–30 µg) from mouse tissues or cell lysates were separated on 12% SDS-PAGE gels and transferred to PVDF membranes using the Trans-Blot Turbo system. Membranes were blocked with 5% non-fat dry milk in TBST and subsequently washed three times in TBST after incubation with primary antibodies (diluted in 5% BSA in TBST) and secondary antibodies (diluted in 5% non-fat dry milk in TBST). Protein detection was carried out using the SuperSignal™ West Pico PLUS Chemiluminescent Substrate ECL kit (34578, Thermo Fisher) or SuperSignal™ West Femto Maximum Sensitivity Substrate kit (34096, Thermo Fisher). Signals were visualized using the Fusion pulse imaging system. The antibodies used are shown in Table 2.

**Table 2:** Antibodies used in this study.

Antibody	Manufacturer
α-ERK	Cell Signaling Technology; catalog no.: 4695
α-p(TEY) ERK	Cell Signaling Technology; catalog no.: 4370
α-p(T207/188) ERK	Kinexus; catalog no.: pk865
α-GAPDH	Thermo Fisher Scientific; catalog no.: MA515738
α-MEK1/2	Cell Signaling Technology; catalog no.: 8727
α-pMEK1/2	Cell Signaling Technology; catalog no.: 9154
α-Upp1	Cell Signaling Technology; catalog no.: 48789
α-PDPN	Abcam; catalog no.: ab256559
α-DUSP6	Abclonal; catalog no.:A0133

### Cloning and plasmids

The 3X-flag ERK1^WT^ cDNA in the pCEFL vector was constructed by Bio Basic Inc. Site-directed mutagenesis was then performed to introduce the R84S and R84H mutations, resulting in the construction of 3X-flag ERK1^R84S^ and 3X-flag ERK1^R84H^ plasmids in pCEFL. The resulting plasmids were subsequently used for transfection and further experiments.

### Cell culture procedures and foci assay

NIH3T3 cells were cultured in Dulbecco’s modified Eagle’s medium (DMEM; Sigma–Aldrich) supplemented with 10% fetal bovine serum (FBS), sodium pyruvate, penicillin-streptomycin, and plasmocin (Biological Industries). Cells were maintained at 37 °C with 5% CO_2_, and routinely tested for mycoplasma contamination. Transfections were performed using TurboFect transfection reagent (Thermo Scientific; catalog no. R0533) according to the manufacturer’s protocol. Approximately 24 h post-transfection, cells were subjected to selection with 400 μg/mL G-418 (Sigma–Aldrich; catalog no. A1720). When applicable, inhibitors were also introduced 24 h post-transfection. Cells were harvested at the indicated time points using 1× SB buffer.

To establish stable cell lines, single *foci* were isolated from mutant-transfected cells, while a pool of cells was collected from those expressing Erk1^WT^, four weeks post-transfection. For the *foci* assay, four weeks after transfection, cells were washed with PBS, fixed with 100% methanol for 20 min, and stained with 4% crystal violet for 5 min, followed by appropriate washing steps.

For experiments involving cancer-derived cell lines, 1 million cells were seeded, and 24 h post seeding, cells were treated with either fresh medium or medium containing DMSO, or 5, 10, or 24 µM BVD-523, or 50 ng/mL EGF (PeproTech; catalog no. AF-100-15). Inhibitors were added for 2 h and EGF for 10 min.

### Protein purification and in vitro kinase assay

Erk proteins used for in vitro kinase assays were expressed in and purified from *E. coli* cells as previously described [45]. In vitro kinase assay and autophosphorylation assay were performed as described previously [44, 46].

Briefly, for the in vitro kinase assay, Erk1^WT^, Erk1^R84S^, Erk1^R84H^, Erk2^WT^, Erk2^R65S^ or Erk2^R65H^ were incubated with myelin basic protein (MBP) and ^32^p[γ]ATP with or without prior incubation with MEK1^EE^ as described for each experiment. For checking sensitivity of inhibitors kinases assays were performed in the presence of increasing concentrations of SCH772984, GDC0994 or BVD523. Level of 32p incorporated into MBP were monitored in β-counter, while incorporation to Erk molecules (autophosphorylation) was determined by exposing to molecules to phosphor-imager following SDS-PAGE. All experiments were conducted at least 3 times in triplicates. The results represent the average values.

### Mass spectrometry

#### Sample preparation

1.2 million cells were seeded in 10 cm tissue culture plate. At the indicated time point, cells were scrapped with 200 μL PBS on ice and flash frozen. Subsequently, cell pellets were lysed with 5% SDS in 50 mM Tris-HCl. Lysates were incubated at 96 °C for 5 min, followed by six cycles of 30 s of sonication (Bioruptor Pico, Diagenode, USA). Protein concentration was determined using the BCA assay (Thermo Scientific, USA) and a total of 120 μg protein was reduced with 5 mM dithiothreitol and alkylated with 10 mM iodoacetamide in the dark. Phosphoric acid was added to the lysates to a final concentration of 1.2%. 90:10% methanol/50 mM ammonium bicarbonate and then added to the samples. Each sample was then loaded onto S-Trap microcolumns (Protifi, USA). Samples were then digested with trypsin (1:50 trypsin/protein) for 1.5 h at 47 °C. The digested peptides were eluted using 50 mM ammonium bicarbonate; trypsin was added to this fraction and incubated overnight at 37 °C. Two more elutions were made using 0.2% formic acid and 0.2% formic acid in 50% acetonitrile. The three elutions were pooled together. An aliquot corresponding to 20 μg protein digest from each sample was set aside for total proteome analysis, while the remaining volume was used for phosphopeptide enrichment. Samples were vacuum-centrifuged to dryness and kept at −80 °C until analysis.

#### Phosphopeptide enrichment

Phosphopeptides were enriched from the total protein digest using a ProPac IMAC-10 column (4 × 50 mm) (Thermo Scientific, P.N. 063276). Loading buffer (A)-0.1% TFA, 30% ACN. Elution buffer(B)- 0.3% NH4OH, pH 11.7. Peptides were loaded on the column in 10 min with flow 0.1 ml/min (0% B). Phosphopeptides were eluted from the column using the following gradient: first elution from 10 to 15 min with flow 0.6 ml/min (B from 0% to 15%), second elution from 15 min to 44 min with flow 0.02 ml/min (B from 15% to 30%), washing from 44 min to 50 min with flow 1 ml/min (B from 30% to 50%) and following equilibration from 50 min to 60 min with flow 1 ml/min (0%B). The phosphopeptides containing peak (second elution fraction) was collected in 1.5 ml reaction vessels (~1,2 ml), vacuum-centrifuged to dryness, and reconstituted in 25 µL in 97:3 acetonitrile: water + 0.1% formic acid.

#### Liquid chromatography

ULC/MS grade solvents were used for all chromatographic steps. Dry peptides were reconstituted in 25 µL in 97:3 acetonitrile: water + 0.1% formic acid. Each sample was loaded using split-less nano-Ultra Performance Liquid Chromatography (10 kpsi nanoAcquity; Waters, Milford, MA, USA). The mobile phase was: A) H2O + 0.1% formic acid and B) acetonitrile +0.1% formic acid. Desalting of the samples was performed online using a reversed-phase Symmetry C18 trapping column (180 µm internal diameter, 20 mm length, 5 µm particle size; Waters). The peptides were then separated using a T3 HSS nano-column (75 µm internal diameter, 250 mm length, 1.8 µm particle size; Waters) at 0.35 µL/min. For global proteomics, peptides were eluted from the column into the mass spectrometer using the following gradient: 4% to 27%B in 165 min, 27% to 90%B in 5 min, maintained at 90% for 5 min and then back to initial conditions.

For total phosphoproteomics, peptides were eluted from the column into the mass spectrometer using the following gradient: 4% to 22%B in 165 min, 22% to 90%B in 5 min, maintained at 90% for 5 min and then back to initial conditions.

#### Mass spectrometry

The nanoUPLC was coupled online through a nanoESI emitter (10 μm tip; New Objective; Woburn, MA, USA) to a quadrupole orbitrap mass spectrometer (Q Exactive HFX, Thermo Scientific) using a FlexIon nanospray apparatus (Proxeon).

Data was acquired in data dependent acquisition (DDA) mode, using a Top10 method. For global proteomics MS1 resolution was set to 120,000 (at 200 m/z), mass range of 375–1650 m/z, AGC of 3e6 and maximum injection time was set to 60msec. MS2 resolution was set to 15,000, quadrupole isolation 1.7 m/z, AGC of 1e5, dynamic exclusion of 45 sec and maximum injection time of 60 msec. For phosphoproteomics similar parameters were used with the exception of MS2 maximum injection time of 150 msec.

#### Data processing and analysis

Raw data was processed with MaxQuant v1.6.6.0 [48]. The data was searched with the Andromeda search engine against the mouse (*Mus musculus*) protein databases as downloaded from Uniprot (www.uniprot.com), appended with common lab protein contaminants and the recombinant human WT or mutant ERK1 sequences. Enzyme specificity was set to trypsin and up to two missed cleavages were allowed. Fixed modification was set to carbamidomethylation of cysteines. For global proteomics variable modifications were set to oxidation of methionines, asparagine and glutamine deamidation, and protein N-terminal acetylation. For phosphoproteomics, phosphorylation of S, T, or Y were additionally included as variable modifications. Peptide precursor ions were searched with a maximum mass deviation of 4.5 ppm and fragment ions with a maximum mass deviation of 20 ppm. Peptide and protein identifications were filtered at an FDR of 1% using the decoy database strategy (MaxQuant’s “Revert” module). The minimal peptide length was 7 amino-acids and the minimum Andromeda score for modified peptides was 40. Peptide identifications were propagated across samples using the match-between-runs option checked. Searches were performed with the label-free quantification option selected. Decoy hits were filtered out.

In total, 5467 proteins and 17,383 phosphorylation sites (across 3789 proteins) were identified and quantified. Differential expression analysis was conducted using the Limma package in R [49], following logarithmic transformation of the data. Missing values were imputed with a bounded minimum value. Differentially expressed proteins were identified using a cutoff of absolute fold change >2 and p-value < 0.05, with a requirement of at least two peptides detected by mass spectrometry.

Unsupervised analysis was employed to identify patterns of protein expression via clustering of differentially expressed proteins and phosphosites. K-Means clustering was performed using standardized log2 normalized intensities. All clustering analyses were executed using RStudio v4.2.2 (RStudio Team (2020). RStudio: Integrated Development for R. RStudio, PBC, Boston, MA URL http://www.rstudio.com/). Principal Component Analysis (PCA) and hierarchical clustering (using Pearson’s dissimilarity and Ward’s method) were conducted based on 4210 proteins with at least two quantified peptides and only valid measurements.

Pathway analysis was performed with Ingenuity Pathway Analysis (IPA) (www.ingenuity.com) to identify significantly relevant biological functions and pathways. Additionally, Kinase Set Enrichment Analysis (KSEA) [50, 51] was applied using the Phosphomatics online tool [52].

### Statistical analysis

Data are presented as mean ± SEM. Statistical significance of the differences between groups was determined by unpaired two-tailed t-test with the GraphPad prism software, San Diego, CA, USA (*P < 0.05, **P < 0.01, ***P < 0.001, ****P < 0.0001).

## Results

### Establishment of an in vivo model to check oncogenicity of Erk per se: a transgenic mouse that allows expression of Erk1^R84H^ in temporally controlled and tissue-specific manners

**Fig. 1:**
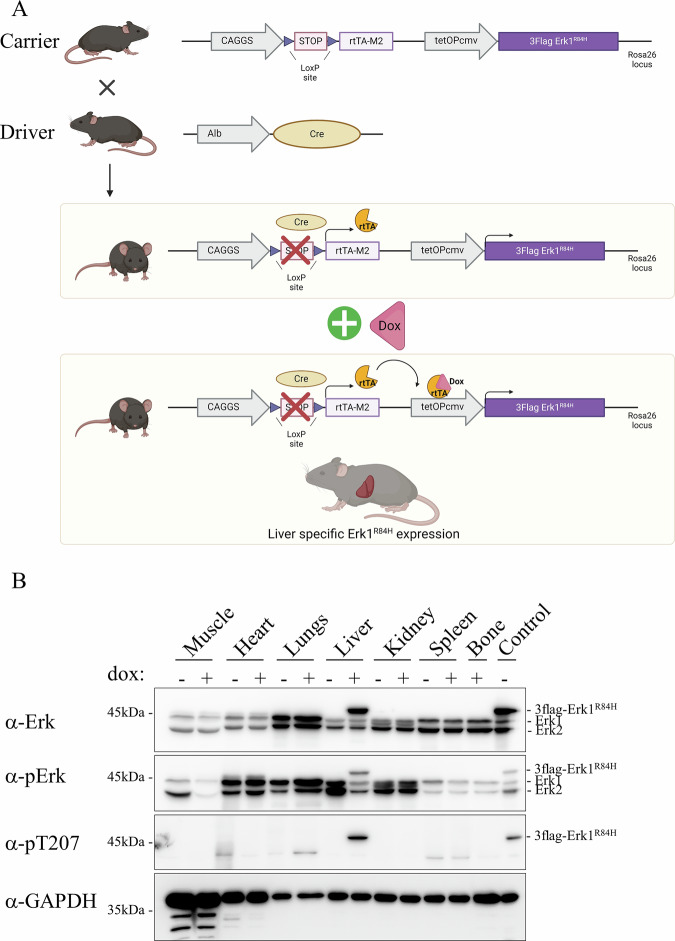
Design, establishment and confirmation of the transgenic mouse model that inducibly expresses Erk1^R84H^ specifically in the liver. **A** Schematic representation of the transgenic mouse model. The upper part of the panel shows the two expression cassettes inserted at the Rosa26 locus of the carrier mice. In the cassette on the left the rtTA coding sequence (rtTA-M2) is transcriptionally controlled by the strong and constitutively active CAGGS promoter, but a transcription intervening sequence (STOP), that is bordered by flox sequences, separates between the promoter and the gene. Therefore, the gene encoding the rtTA-M2 could be transcribed only if the “STOP” is removed. In the cassette on the right the cDNA encoding flag-tagged Erk1^R84H^ could be transcribed under the tetO Pcmv-promoter, which is activated by rtTA-M2. The second row of the panel shows the driver mouse in which Cre recombinase is expressed only in the liver, under the albumin promoter. In progeny of crossbreeding between the carrier mice and the Alb-Cre driver mice (middle panel), termed hepatic-hetero-Erk1^R84H^ mice, the liver-specific expression of Cre recombinase causes excision of the STOP sequence in hepatocytes only and consequently transcription of rtTA-M2. Upon doxycycline (dox) administration (bottom panel), dox-bound rtTA-M2 is active as a transcriptional activator and activates the tetO Pcmv-promoter that drives expression of 3xFlag-tagged Erk1^R84H^ specifically in the liver. **B** Western blots analysis, with the indicated antibodies, of protein lysates prepared from various organs of hepatic-hetero-Erk1^R84H^ mice fed with dox-supplemented diet for 2 weeks.

To check whether expression of Erk1^R84H^ may give rise to tumors in vivo, we established a transgenic mouse model that allows tissue-specific and temporally controlled expression of Erk1^R84H^. While the model allows expression in any tissue, this study is focused on the liver. The RTK-Ras-Raf-MEK-Erk pathway is abnormally active in 30% of liver cancer cases and in ~80% of liver cancer in mice induced by diethylnitrosamine (DEN) [4, 48], suggesting that the liver is a suitable tissue for testing whether activation of Erk alone could cause cancer. The design of the transgenic system that allows liver-specific expression when doxycycline (dox) is provided in the diet, is described in detail in Supplementary File S1 and in Fig. 1A. Mice harboring one allele of the expression cassette were termed hepatic-hetero-Erk1^R84H^, while those harboring two alleles were termed hepatic-homo-Erk1^R84H^ mice. The levels of Erk, pErk and pT207 were compared between the hepatic-hetero-Erk1^R84H^ and the hepatic-homo-Erk1^R84H^ mice (Supplementary Fig. S1).

To check the operability of the model, hepatic-hetero-Erk1^R84H^ mice were fed with a dox-supplemented diet for 2 weeks, and various organs were collected to check for Erk1^R84H^ expression. Expression of Erk1^R84H^ was observed only in the liver, and only in mice provided with dox-supplemented diet, validating the model design (Fig. 1B). Importantly, the transgenic Erk1^R84H^ protein was phosphorylated on the TEY motif, demonstrating that in the context of the whole organism it maintained the property of becoming spontaneously active. Notably, the phosphorylation level was not very high (Fig. 1B, row 2), as a consequence of powerful feedback inhibition as will be explained in detail below. As expected from in vitro and tissue cultures studies [44, 46], Erk1^R84H^ was also autophosphorylated on Thr207 in the liver (Fig. 1B, row 3), a phosphorylation reported to be associated with human diseases [49]. To our knowledge, this is the first model that allows activation of Erk molecules per se (i.e, without activating upstream components) in vivo, in tissue-specific and temporally controlled manners.

### Liver-specific expression of Erk1^R84H^ causes development of hepatocellular carcinoma (HCC)

**Fig. 2:**
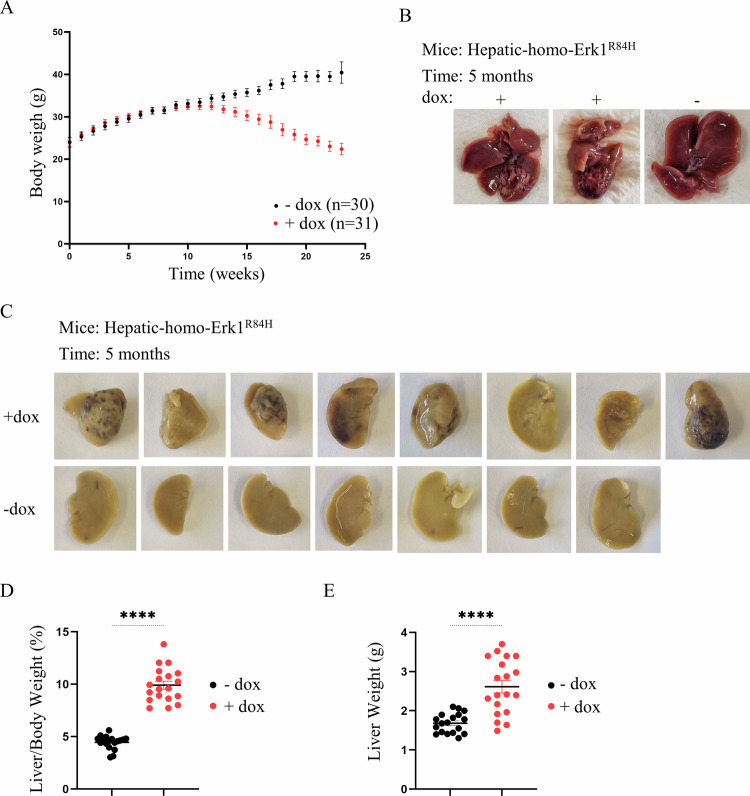
Liver-specific Erk1^R84H^ expression causes hepatocellular carcinoma (HCC). **A** Changes in total body weight of hepatic-Erk1^R84H^ mice fed with a regular diet (black circles; *n* = 31) or dox-supplemented diet (red circles; *n* = 30). Data are shown as mean ± SEM. **B** Macroscopic appearance of livers from hepatic-homo-Erk1^R84H^ mice provided with dox-supplemented diet for 5 months (two photos on the left) showing signs of significant deformations, and of a hepatic-homo-Erk1^R84H^ mouse provided with regular diet for the same time (photo on the right). **C** As in B but after tissue fixation. **D** Liver-to-body weight ratio in hepatic-homo-Erk1^R84H^ mice provided with a regular diet (black circles) or a dox-supplemented diet (for inducing liver-specific expression of Erk1^R84H^) (red circles). Data are represented as mean ± SEM. Statistical significance was determined by unpaired two-tailed t test with significance indicated by ****, representing a *P*-value of *p* < 0.0001. **E** Total liver weight of hepatic-homo-Erk1^R84H^ mice fed dox-supplemented diet (red circles) or regular diet (black circles). Data are represented as mean ± SEM. Statistical significance was determined by unpaired two-tailed t test with significance indicated by ****, representing a *P*-value of *p* < 0.0001. Notably, investigators were not blinded to the experimental groups.

To assess whether expression of Erk1^R84H^ affects the liver in any way, groups of hepatic-hetero-Erk1^R84H^ and hepatic-homo-Erk1^R84H^ mice were provided with a dox-supplemented diet (to induce liver-specific Erk1^R84H^ expression) while other groups of the same mice were provided with a regular diet. Mice were randomly selected for each group. It was noted that about 12 weeks after the beginning of the experiment both hepatic-hetero-Erk1^R84H^ and hepatic-homo-Erk1^R84H^ mice provided with dox-supplemented diet began to lose weight and continued to lose weight until the end of the experiment (Fig. 2A). During the course of the experiment mice were sacrificed at 111, 152, and 182 days (3.7, 5 and 6 months respectively) after change of diet, and the livers were removed for analysis.

Inspection of the livers of hepatic-homo-Erk1^R84H^ mice provided with dox-supplemented diet for 5 months revealed dramatic deformation, including areas of discoloration and nodular growths, which are suggestive of tumor-like bodies (Fig. 2B, C). There was a significant portion of the liver that looked darker, possibly indicating areas of necrosis or hemorrhage. The surface of the liver was not uniform, but rather had multiple swollen areas which could be indicative of lesions or tumors. These irregularities were in stark contrast to the smooth, homogenous and overall normal appearance of the livers removed at the same timepoints from similar mice not expressing the active Erk1 (Fig. 2B, C). Also, at all timepoints the liver to body weight ratio of hepatic-homo-Erk1^R84H^ was markedly higher compared to that in mice not expressing it (Fig. 2D). The mean of liver weight of mice not expressing Erk1^R84H^ was 1.68+/− 0.24 mg while the liver weight mean of mice expressing Erk1^R84H^ was 2.61 +/− 0.68 mg (Fig. 2E).

**Fig. 3:**
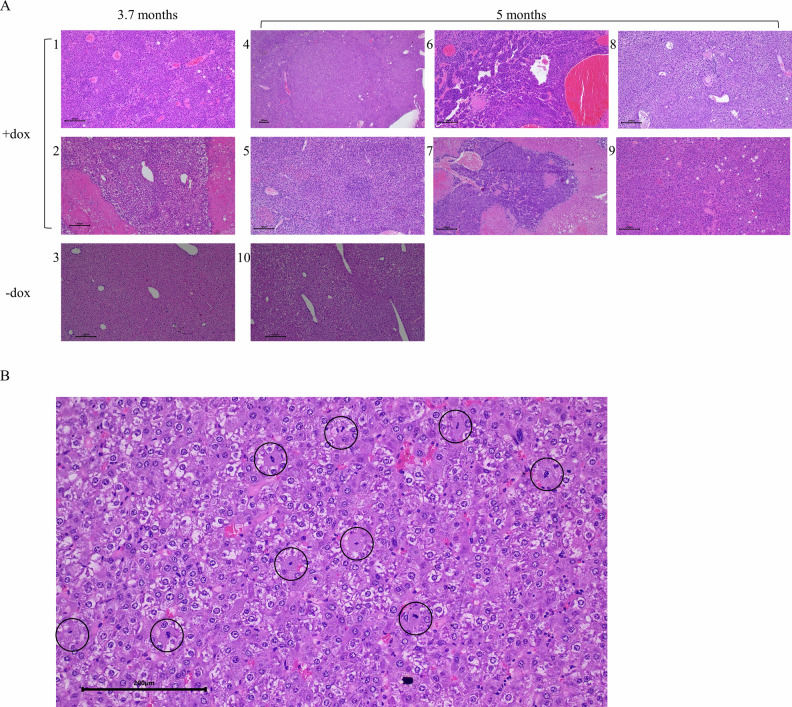
Pathological analysis of livers from hepatic-Erk1^R84H^ transgenic mice reveals tumor development and hepatic abnormalities. **A** Representative H&E staining of liver cross sections from hepatic-homo Erk1^R84H^ mice provided with dox-supplemented diet for 3.7 months (1–2) or 5 months (4–9), or with regular diet for the same periods (3 and 10) (scale bar = 200 µM). **B** An enlarged view of a liver section from a hepatic-homo mouse expressing Erk1^R84H^ for 5 months, highlighting increased mitotic activity (reflected by chromosomes at various stages of mitosis; circled regions) as a marker of uncontrolled cell proliferation, as is common in HCC (scale bar = 200 µM).

Pathological examination of hematoxylin and eosin (H&E)-stained cross sections revealed that liver was seriously affected in 100% of both heterozygous or homozygous, upon induction of Erk1^R84H^. Notable alterations in livers of hepatic-homo-Erk1^R84H^ mice expressing the transgene for 111 days (3.7 months) included increased basophilia, pronounced anisokaryosis, and varying cytoplasmic volumes, as illustrated in Fig. 3A1. None of these phenotypes were observed in livers of identical hepatic-homo-Erk1^R84H^ mice not expressing Erk1^R84H^ (Fig. 3A3). Additionally, livers expressing Erk1^R84H^ exhibited heterogeneity in cellular density and occasional presence of deeply eosinophilic cytoplasmic inclusions, as well as areas of coagulation necrosis (Fig. 3A2). These phenotypes suggest potential liver dysfunction.

Pathological analysis of livers removed from hepatic-homo-Erk1^R84H^ mice expressing Erk1^R84H^ for more than 5 months revealed an increased tendency for tumor development. Specifically, 54.5% of the livers (6 out of 11) exhibited lesions ranging from moderately to poorly differentiated hepatocellular carcinoma (HCC) (Fig. 3A4-9). Liver sections prepared from these mice showed an increased mitosis rate (Fig. 3B), irregular *foci* of proliferation (Fig. 3A4, A5), and areas of hemorrhage and coagulation necrosis (Fig. 3A6, A7). These phenotypes were not observed in liver sections from hepatic-homo-Erk1^R84H^ mice fed a regular diet (i. e. not expressing Erk1^R84H^) for the same period (Fig. 3A10). The 5 mice that did not manifest clear HCC phenotypes displayed anisokaryosis (Fig. 3A8) and accumulation of eosinophilic cytoplasmic globules (Fig. 3A9), similar to the phenotype manifested by the mice expressing Erk1^R84H^ for 3.7 months.

The phenotype of hepatic-hetero-Erk1^R84H^ mice that were on a dox-supplemented diet for about 5 months was less severe than that of the hepatic-homo-Erk1^R84H^ mice with respect to tumor development. Nevertheless, they did demonstrate an inclination towards dilation of arteries, suggesting a problem with blood pressure (Supplementary Fig. S2), and exhibited similar phenotypes to those seen in hepatic-homo-Erk1^R84H^ mice with respect to accumulation of eosinophilic globules and anisokaryosis (Supplementary Fig. S2A, B). It appears that hepatic-hetero-Erk1^R84H^ mice fed on a dox-supplemented diet represent a stage where the tissue is inclined or prone to become cancerous, while many of the hepatic-homo-Erk1^R84H^ mice, having two alleles of Erk1^R84H^ develop the full-scale disease. Importantly, liver disease was significant and prominent in 100% of the mice expressing the active Erk1^R84H^, whether heterozygous or homozygous, compared to 0% among those fed with a regular diet and therefore did not express the active Erk1^R84H^. The difference between heterozygous and homozygous expression is expected, as noted in other mouse models that used the same double-cassette expression system [50, 51] (see discussion).

### Kidneys are also significantly affected in mice expressing Erk1^R84H^ in the liver

**Fig. 4:**
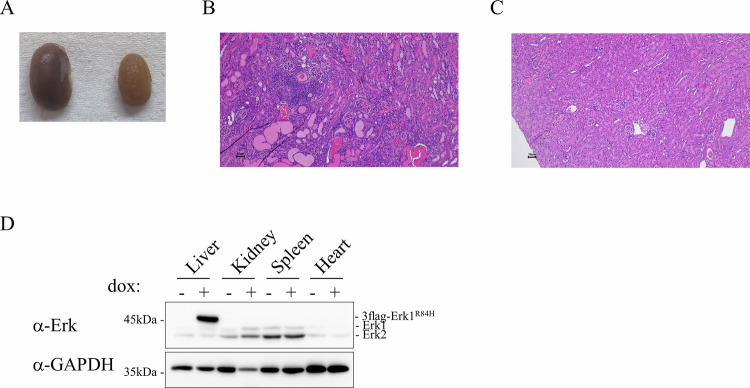
Kidney are altered in hepatic-Erk1^R84H^ mice although Erk1^R84H^ is expressed exclusively in the liver. **A** Macroscopic morphology of kidneys of hepatic-homo-Erk1^R84H^ mouse expressing (right) or not expressing (left) Erk1^R84H^ for 3 months. **B** Representative H&E-stained section of kidneys from hepatic-homo Erk1^R84H^ mice supplemented with dox-supplemented diet for 3 months showing kidney abnormalities. **C** As in (**B**) but sections from a control mouse, fed with regular diet, showing a normal kidney appearance. **D** A western blot analysis of protein lysates from various organs of hepatic-homo-Erk1^R84H^ mouse expressing Erk1^R84H^ for 5 months. Note that Erk1^R84H^ expression was restricted to the liver. No Erk1^R84H^ expression was detected in the kidney.

While removing the livers of mice expressing Erk1^R84H^ we noted the abnormal appearance of the kidney. Kidneys were smaller in size and somewhat paler compared to those from mice not expressing the active Erk1^R84H^ variant (Fig. 4A). We thus examined kidneys of homozygous mice expressing Erk1^R84H^ for 3 months. The pathologist’s report highlighted several renal pathologies, including the presence of protein casts, glomerular thickening, and perivascular infiltration (Fig. 4B), phenotypes that were not observed in a similar mouse not expressing Erk1^R84H^ in the liver (Fig. 4C). These findings suggest a serious renal disorder causing both functional and structural kidney alterations.

The renal changes are probably a secondary effect of hepatic dysfunction caused by the specific expression of Erk1^R84H^ in the liver (Supplementary Fig. S3). This conclusion is based on the analysis of serum markers indicative of both hepatic and renal function, which revealed that liver-associated markers (e.g., albumin, bilirubin, total protein, calcium) were altered at both early and late time points, whereas kidney-associated markers (e.g., urea and creatinine) showed changes only at later time points (Supplementary Fig. S3). The protein casts in the kidneys, which represent protein accumulation, might originate from protein accumulation in the liver, implying a deficiency in hepatic protein processing and subsequent accumulation in the kidneys. Importantly, despite the dramatic renal effects observed, we confirmed that Erk1^R84H^ expression was confined to the liver, with no detectable expression in the kidneys – even in mice expressing Erk1^R84H^ for 5 months, as indicated by α-Erk antibody (Fig. 4D). The temporal pattern of serum marker changes, combined with the absence of Erk1^R84H^ expression in the kidneys, supports the hypothesis that the renal pathology arises as a secondary consequence of sustained liver dysfunction.

### TEY phosphorylation of Erk1^R84H^ is shut off during the development of liver cancer

**Fig. 5:**
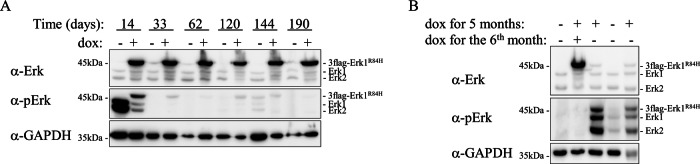
Dramatic downregulation of TEY phosphorylation of Erk1^R84H^ and endogenous Erk1/2 during the development of liver cancer. **A** Western blot analysis of liver protein lysates from hepatic-Erk1^R84H^ mice provided with dox-supplemented diet for the indicated time periods. **B** Western blot analysis of livers of hepatic-Erk1^R84H^ mice grown on regular diet (1^st^ and 4^th^ lanes from the left), or dox-supplemented diet (2^nd^ lane from left) for six months, or on dox-supplemented diet for 5 months and then on regular diet for 1 month (3^rd^ and 5^th^ lanes from the left).

To check for the linkage between the activity of Erk1^R84H^ and oncogenic transformation of the liver, we followed the status of Erk1^R84H^ phosphorylation for 190 days. Similar to the case in the experiment shown in Fig. 1B, in this experiment too, TEY was highly phosphorylated in mice expressing Erk1^R84H^ for 14 days. However, the phosphorylation levels were significantly lower as early as 33 days after induction of expression and remained at the edge of detectability throughout the experiment (Fig. 5A). Namely, as liver pathology progressed, Erk1^R84H^’s phosphorylation became extremely low, and remained barely detectable in the cancerous liver (Fig. 5A). Notably, phosphorylation of endogenous Erk1 and Erk2 molecules was also downregulated (Fig. 5A, row 2). Similar kinetics of pErk1^R84H^ downregulation were observed in the hepatic-homo-Erk1^R84H^ model (Supplementary Fig. S4), further supporting the generality of this phenomenon. These findings suggest that tumors are maintained and continue to grow despite the markedly low catalytic activity of the transforming oncogene.

We next asked whether the downregulation in TEY phosphorylation in the hepatic-Erk1^R84H^ mouse model is a consequence of a new biochemical activity that emerged in the transformed hepatocyte or is still dependent on the activity of Erk1^R84H^. In other words, would the vanished TEY phosphorylation re-appear if Erk1^R84H^ expression would be shut-off? To explore this question, we used hepatic-hetero-Erk1^R84H^ mice expressing Erk1^R84H^ for 5 months, replaced the dox-supplemented diet with a regular diet and sacrificed the mice one month later. As is shown in Fig. 5B, while expression of Erk1^R84H^ was downregulated upon transfer to a regular diet, TEY phosphorylation of both Erk1^R84H^ and endogenous Erk1/2 was concomitantly elevated (Fig. 5B, columns 3 & 5, row 2). This observation underscores the reliability of our transgenic mouse model and strongly suggests that the downregulation of TEY phosphorylation of Erk1^R84H^ is a direct consequence of its own activity.

### In the process of transforming NIH3T3 cells, phosphorylation of the oncoprotein Erk1^R84H^ is dramatically reduced and becomes undetectable in the transformed cells

**Fig. 6:**
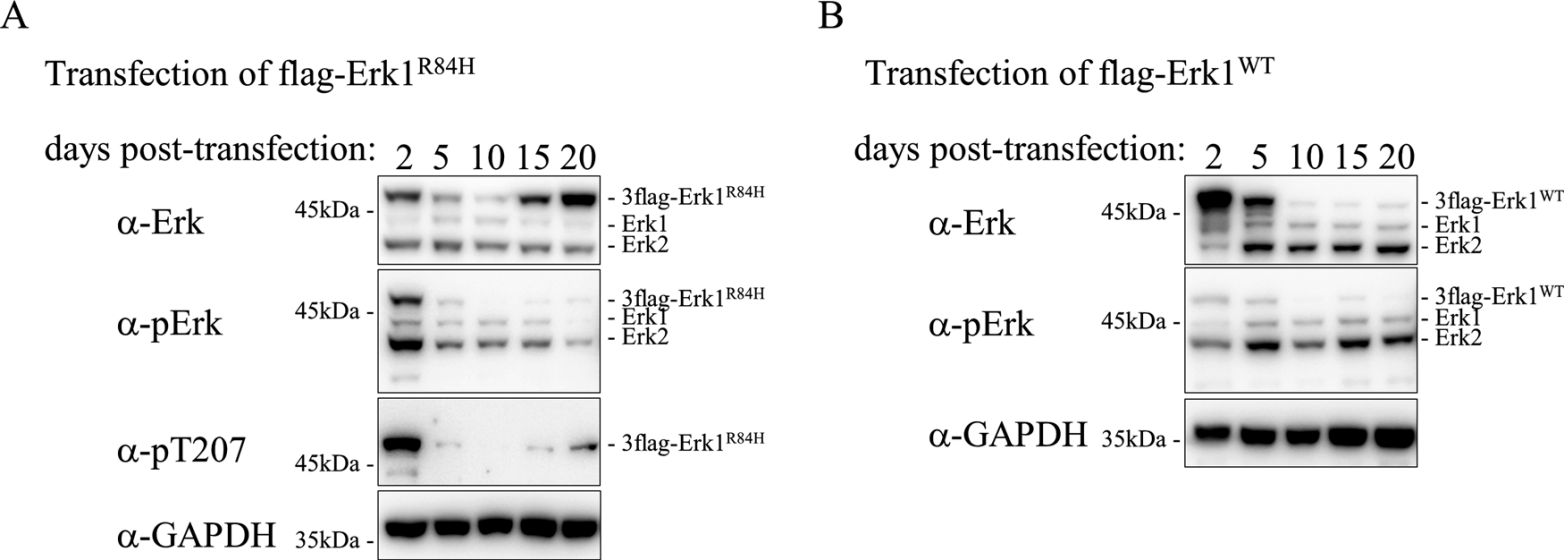
Oncogenic transformation of NIH3T3 cells by Erk1^R84H^ is also accompanied by downregulation of TEY phosphorylation. Western blot analysis of lysates prepared from NIH3T3 cells transfected with a plasmid expressing 3Xflagged-Erk1^R84H^ (**A**) or with 3Xflagged-Erk1^WT^ (**B**) at the indicated timepoints following transfection.

To explore the oncogenic mechanism utilized by Erk1^R84H^, to test whether it is sensitive to Erk inhibitors and to explore the shut-off mechanism, we used NIH3T3 cells, which can be transformed by this molecule [44]. We first followed the status of Erk1^R84H^ in cells transfected with a plasmid expressing 3Xflagged-Erk1^R84H^. A plasmid expressing 3Xflagged-Erk1^WT^ was transfected in parallel as a control. G418 selection was imposed 24 h post transfection and samples of cells were harvested at 2, 5, 10, 15 and 20 days post transfection. Western blot analysis showed that Erk1^R84H^ was catalytically active, as manifested by its strong phosphorylation on the TEY motif at day 2 post transfection. It remained phosphorylated, though to lower levels, for 5 days after transfection (Fig. 6A, row 2). Phosphorylation level of Erk1^WT^ was low at these timepoints (Fig. 6B, second row). As expected, due to the G418 selection process and death of cells not harboring the plasmids, from day 5 onward Erk1^R84H^ expression levels decreased, and accordingly, in day 10 phosphorylation was almost undetectable (Fig. 6A, rows 1 & 2). While expression of Erk1^R84H^ was clearly observed at days 15 and 20 post-transfection (as at this timepoint most cells are G418-resistant, i.e., harboring the expressing plasmid) its phosphorylation was almost undetectable (Fig. 6A). Phosphorylation of Thr207 was also significantly downregulated from day 2 to day 20 (Fig. 6A, row 3). These intriguing results are reminiscent of the observation in the hepatic-Erk1^R84H^ mouse model, as between day 15 and 20 post transfection, when Erk1^R84H^ phosphorylation is barely detectable, transformed *foci* develop in the plate.

Unlike the case of Erk1^R84H^, expression of Erk1^WT^, which was reduced at day 10, did not recover, and remained low at day 20 (Fig. 6B, first row). As explained, Thr-207 phosphorylation is exclusive to active variants and therefore not detected in cells transfected with a vector that drives Erk1^WT^ expression.

**Fig. 7:**
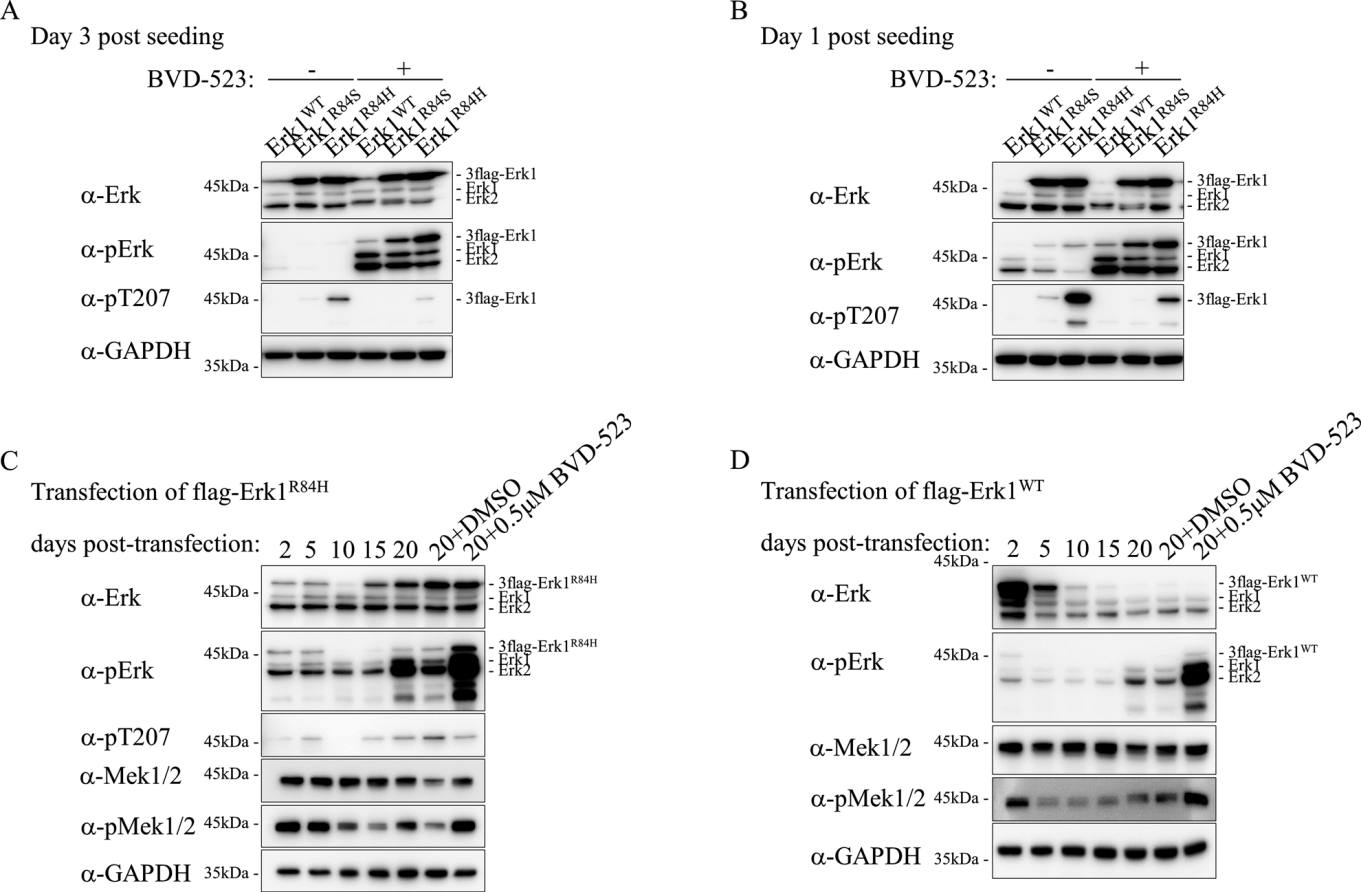
Suppression of Erk1^R84H^ phosphorylation occurs via a powerful feedback mechanism as it is relieved by Erk inhibitors. **A** Western blot analysis of protein lysates prepared from stable NIH3T3 clones expressing Erk1^WT^, Erk1^R84S^, or Erk1^R84H^ 3 days post plating of frozen cells, without (left three lanes) or with (right three lanes) exposure to 5 μM BVD-523 for 2 h. **B** As in A but lysates were prepared from cells 24 h post plating of frozen cells. Western blot analysis of lysates prepared from NIH3T3 cells transfected with a plasmid expressing Erk1^R84H^ (**C**) or Erk1^WT^ (**D**) at the indicated timepoints post transfection (5 lanes from the left), and of cultures provided with 5 μM BVD-523 or DMSO for 2 h at day 20 post transfection (the two lanes at the right).

As in the hepatic-Erk1^R84H^ mouse model Erks’ phosphorylation was almost undetectable in the tumors themselves, we also examined its phosphorylation in stable clones expressing Erk1^WT^ or Erk1^R84H^. In this experiment we also included cells transformed with Erk1^R84S^ (note that clones expressing Erk1^WT^ are derived from G418-resistant colonies while clones expressing Erk1^R84S^ or Erk1^R84H^ are derived from *foci*). Notably, clones expressing Erk1^R84H^ or Erk1^R84S^ remained transformed following 5 passages or more, as well as following freezing and thawing steps, continuing to exhibit altered cell morphology and enhanced growth rate. As reported previously, a clear evidence for the maintenance of the transformed phenotype is the ability of these cells, after several passages, and taken from frozen stocks, to give rise to tumors in nude mice [46]. Despite their transformed state, levels of TEY phosphorylation of Erk1^R84H^ and Erk1^R84S^ were consistently below detection levels in these clones, although the molecules were clearly expressed (Fig. 7A, three left lanes). Thus, similar to the case in mice, cells transformed by Erk1^R84H^ or Erk1^R84S^ maintain their oncogenically-transformed phenotype despite the markedly low catalytic activity of the transforming oncogene. Intriguingly, not only Erk1^R84H^ and Erk1^R84S^ were not phosphorylated in cells transformed by them, but also phosphorylation of endogenous Erk1/2 was very low in these cells (Fig. 7A, row 2). Thus, in both hepatic-Erk1^R84H^ mice and in NIH3T3 cells, the machinery that suppresses Erk1/2 phosphorylation upon Erk1^R84H^ expression is very efficient, affecting auto-phosphorylated Erk molecules (the active variants), and also those phosphorylated by the RTK-Ras-Raf-MEK cascade (endogenous Erk1/2). In stable clones harboring the vector expressing Erk1^WT^, the observation is puzzling. The exogenous Erk1^WT^ is expressed at a relative low level and is not active (Fig. 7A, lanes 1). However, the phosphorylation of the endogenous Erks is still suppressed, as in cells expressing Erk1^R84H^ and Erk1^R84S^. Perhaps some expression levels of Erk1^WT^, higher than in native levels, are sufficient to activate the machinery that suppresses Erk phosphorylation.

We further monitored TEY phosphorylation at different stages of the life of the stable clones and found that at particular short time-windows in the culture life, when cells are plated sparsely, or when cells are just thawed from a frozen stock (no longer than 24 h after thawing) TEY phosphorylation could be detected. Note three left lanes in Fig. 7B, which shows analysis of a culture 24 h after thawing (compare to three left lanes in 7 A). At all other time Erk TEY phosphorylation is strongly suppressed.

### Suppression of Erk1^R84H^ phosphorylation occurs via a powerful feedback mechanism that is part of the oncogenic process

We next wished to test whether in culture too, phosphorylation of Erk could be restored by suppression of the overactive Erk1^R84H^, and thereby confirming that the potent downregulation of the active form of Erk1^R84H^ is directly linked to its own activity. A possible way of reducing Erk1^R84H^ activity could be through the use of Erk’s pharmacological inhibitors. The rationale is that if it is the high activity of Erk1^R84H^ or Erk1^R84S^ that negatively regulates their own phosphorylation then its inhibition would restore phosphorylation.

This experimental approach is valid, however, only if Erk1^R84H^ and Erk1^R84S^ are proven to be sensitive to Erk inhibitors, which were developed against MEK-activated Erks and not against autoactivating molecules [22, 29, 52, 53]. We thus first performed a series of systematic experiments, in which we found that the oncogenic molecules Erk1^R84H^ and Erk1^R84S^ maintain sensitivity to several Erk-specific inhibitors, including BVD-523 (described in Supplementary File S2).

Having verified that Erk1^R84H^ and Erk1^R84S^ are sensitive to the pharmacological inhibitors (Supplementary File S2), we exposed *foci*-derived clones, stably expressing Erk1^R84H^ or Erk1^R84S^, to BVD-523. This treatment caused strong phosphorylation of the TEY motif of all Erk molecules in the cells (Fig. 7A and 7B, 3 right lanes). The response of Erk molecules was so rapid that within 2 h of treatment the signal was detected (Fig. 7A). While exposure to BVD-523 boosted TEY-phosphorylation, it decreased Thr207 phosphorylation of Erk1^R84H^ (Fig. 7A, B, 3^rd^ row). Given that Thr207 phosphorylation is a consequence of autophosphorylation [44, 46], its downregulation is expected once the catalytic activity of Erk1^R84S^ and Erk1^R84H^ is inhibited.

Exposure to BVD-523 also elevated TEY phosphorylation in stable clones harboring plasmid carrying Erk1^WT^ (Fig. 7A, B, 4^th^ column), suggesting that this molecule also imposes some degree of negative feedback inhibition.

To test whether the strong induction of Erk’s phosphorylation is a general phenomenon upon Erks’ inhibition, and not exclusive to BVD-523, the stable clones were exposed to SCH772984 and GDC0994. Similar to BVD-523, GDC0994 restored Erk’s phosphorylation (Supplementary Fig. S5, columns 4 and 9). SCH772984 did not affect Erk phosphorylation, probably due to its different mechanism of inhibition that interferes with Erk phosphorylation by MEK (see Discussion) (Supplementary Fig. S5, columns 3 and 8).

We next wondered whether the downregulation of Erk observed during the very early oncogenic transformation stages is also mediated via feedback inhibition, as was the case in the stable clones. For that we repeated the transfection experiment, and at day 20, when the shut-off of Erk phosphorylation is apparent, exposed cells to BVD-523 for 2 h. This treatment caused significant elevation of TEY phosphorylation of the Erk1^R84H^ protein, as well as of endogenous Erk1 and Erk2 molecules (Fig. 7C). Thr-207 phosphorylation was not affected (Fig. 7C, row 3). The effect of BVD-523 suggests that the rapid disappearance of TEY phosphorylation in cells expressing Erk1^R84H^ and Erk1^R84S^ is a consequence of the unregulated activity of these molecules, which most probably inhibit pathway’s upstream components and also activates negative regulators such as phosphatases (confirmed by proteomic analysis, see below; see Fig. 11D). Once Erk1^R84H^ and Erk1^WT^ are inhibited by the pharmacological reagents, this feedback inhibition is relieved, leading to elevated phosphorylation of MEKs (Fig. 7C, row 5 and Fig. 7D, row 4) and in turn to MEK-mediated Erk phosphorylation (Fig. 7C, D, row 2). The fact that the feedback inhibition is so profound as early as 20 days after transfection, suggests that Erk1^R84H^ and Erk1^R84S^ inhibition is an early step in the oncogenic sequela.

### Although active Erks are shut-off in the course of oncogenic transformation, Erk inhibitors prevent transformation

**Fig. 8:**
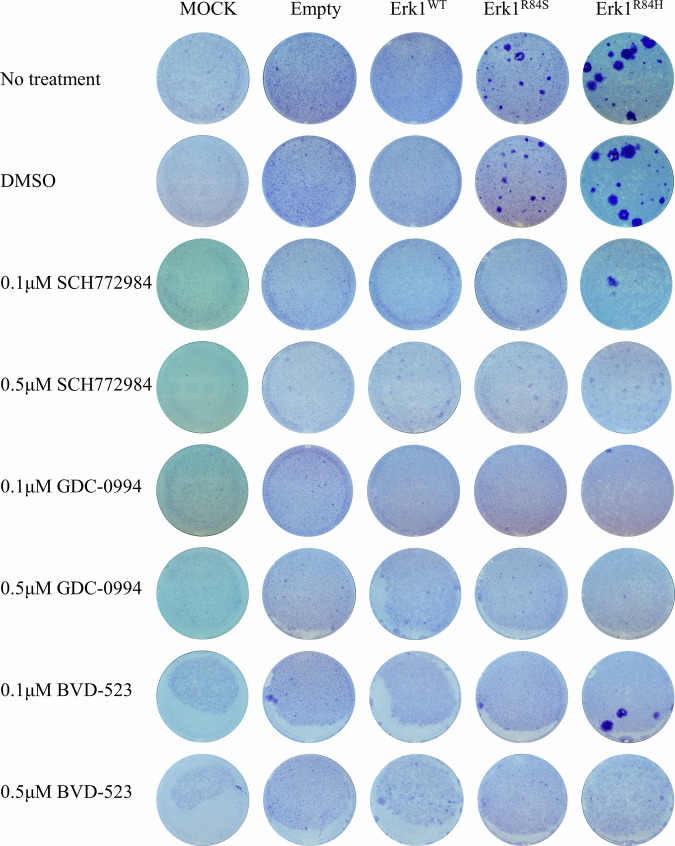
Erks’ pharmacological inhibitors prevent oncogenic transformation of NIH3T3 cells by Erk1^R84S^ and Erk1^R84H^. Crystal violet staining of NIH3T3 cells transfected with plasmids expressing Erk1^WT^, Erk1^R84S^, Erk1^R84H^, or an “empty” plasmid. 24 h post-transfection, cells were subjected to G418 selection. Erk inhibitors were introduced 48 h post-transfection. Staining was performed 30 days post-transfection to assess cell viability and focus formation.

The observation that Erk1^R84S^ and Erk1^R84H^ seem to be almost hermetically shut-off in the course of transformation and remain suppressed when the transformed phenotype is maintained, raises the question of whether their catalytic activity is at all required for the oncogenic process. To address this riddle, we tested the ability of Erk1^R84S^ and Erk1^R84H^ to transform NIH3T3 cells in the presence of inhibitors. NIH3T3 cells were transfected with plasmids expressing Erk1^WT^, Erk1^R84H^, Erk1^R84S^, or with an “empty” plasmid. 24 h after transfection cells were provided with G418 and with 0.1 or 0.5 μM SCH772984, GDC-0994, or BVD-523. Media containing the inhibitors was changed every other day and the cells were monitored for a period of 30 days. In plates not exposed to inhibitors and expressing Erk1^R84H^ or Erk1^R84S^ *foci* appeared as expected (Fig. 8). In plates supplemented with inhibitors, *foci* were commonly not observed, suggesting that inhibiting the catalytic activity of Erk1^R84H^ or Erk1^R84S^ prevented the oncogenic process (Fig. 8). This implies that Erk1^R84S^ and Erk1^R84H^ activity is critical for transformation, even though it is shut-off at early stages of the process. It should be noted that at the concentrations used the inhibitors were not toxic to the cultures and cells of all clones did proliferate and fill the plates. The effect was specific to the formation of *foci* (Fig. 8).

Notably, this experiment disclosed a difference in the response to inhibitors between Erk1^R84S^ and Erk1^R84H^. While 0.1 μM of SCH772984 or BVD-523 was sufficient to completely inhibit the appearance of *foci* in Erk1^R84S^-transfected cells, it was not sufficient to absolutely eliminate *foci* development by Erk1^R84H^ (Fig. 8). It seems that Erk1^R84H^ is a more robust and aggressive onco-protein than Erk1^R84S^.

**Fig. 9:**
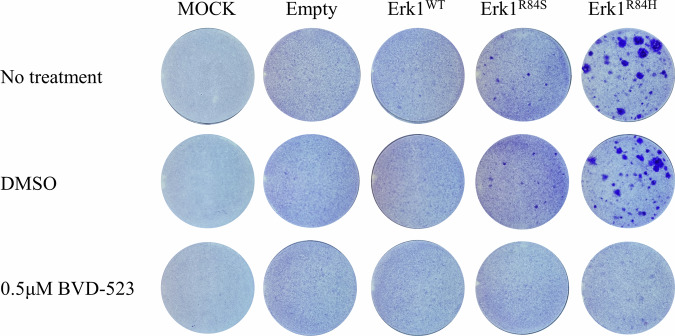
Introduction of Erk inhibitors at a late stage post-transfection stops further development of transformed *foci.* NIH3T3 cells transfected with Erk1^R84H^ were allowed to grow for 13 days until small *foci* were visible, at which point 5 μM of BVD-523 was added to the media. Cultures were stained with crystal violet 20 days post-transfection.

Next, we examined the effect of introducing inhibitors at later stage post-transfection to assess whether relieving the negative feedback could halt or regress tumor progression. 5 μM of BVD-523 was thus added 13 days post-transfection, at a time when small *foci* had already formed. As shown in Fig. 9, the introduction of inhibitors at this later stage effectively blocked further progression of the *foci* (Fig. 9).

### Erk proteins are also shut-off via a negative feedback machinery in cells derived from human cancer

**Fig. 10:**
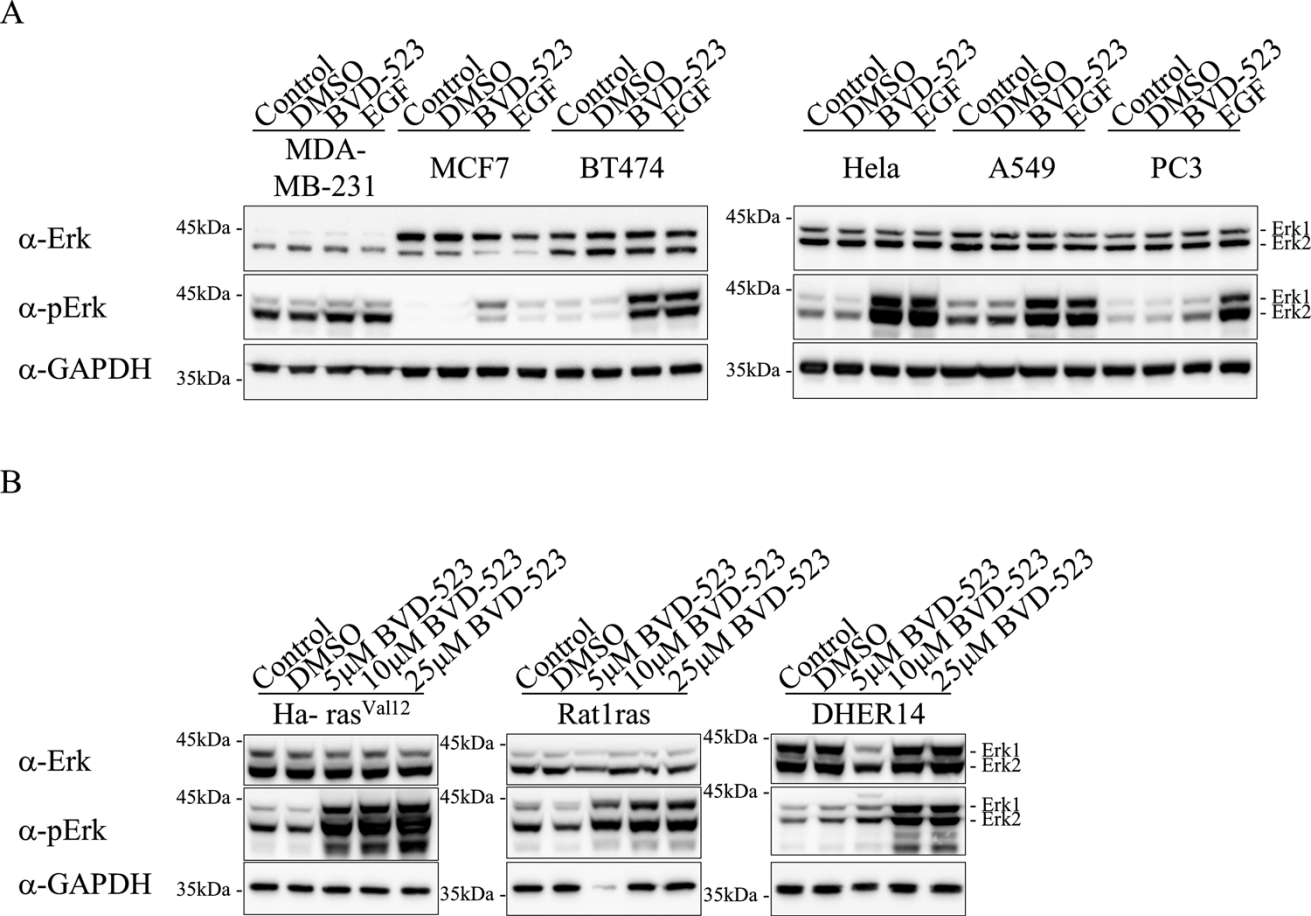
Erk proteins are subject to a strong feedback inhibition in human cancer-derived cell lines and in laboratory models of oncogenic transformation. **A** Western blot analysis of lysates prepared from the indicated cancer-derived cell lines, exposed or not exposed to 5 μM BVD-523 for 2 h or to EGF (a positive control) for 10 min. **B** As in A but using the indicated laboratory-transformed cell lines.

As described in the introduction, various levels of phosphorylated Erks were reported in different cancers [37–41]. These very different levels were proposed to reflect the resultant interplay between activation and feedback inhibition pathways of the RTK-Ras-Raf-MEK-Erk cascade [54–59]. It is not clear, however, whether the dramatic downregulation, in fact, shut-off, of Erks’ activity, as we observed in mice and cells transformed by Erk1^R84S^ is common in cancer. It might be that the phenomenon is a peculiar property of the experimental models or of the Erk1^R84H^ and Erk1^R84S^ proteins. We thus tested a battery of cancer-derived cell lines for Erks’ phosphorylation before and after exposure to Erk inhibitors (Fig. 10A). The cell lines tested included MDA-MB-231 (derived from breast adenocarcinoma), MCF7 (derived from metastatic breast adenocarcinoma), BT474 (derived from solid, invasive ductal breast carcinoma), HeLa (derived from cervical cancer), A549 (derived from non-small cell lung cancer), PC3 (derived from bone metastasis of prostate cancer) and LnCAP (derived from metastatic prostate adenocarcinoma). Of these six cell lines, significant levels of phospho (TEY)-Erk were monitored only in MDA-MB-231 cells (Fig. 10A). In the other cell lines phosphorylation levels were very low, in some barely detectable (e.g., MCF7 and BT474), similar to the situation in cells transformed by Erk1^R84S^ or Erk1^R84H^. Upon exposure to BVD-523, the MCF7, BT474, HeLa and A549 cells manifested high levels of Erk phosphorylation (Fig. 10A). Some elevated phosphorylation was also observed in PC3 cells (Fig. 10A). No change in phosphorylation levels was monitored in MDA-MB-231 cells in which the phosphorylation was a priori high (Fig. 10A). Thus, in five out of the six cell lines tested an efficient Erk-dependent feedback inhibition is operating, leading to a remarkable reduction of its own phosphorylation, in some cases below detection levels. Furthermore, we tested additional cell lines that were oncogenically transformed in the laboratory, including NIH3T3/Ha-ras^Val12^ (NIH3T3 cell line transformed with oncogenic Ha-ras^G12V^), Rat1ras (rat fibroblast cell line transformed with oncogenic Ha-ras^G12V^) and DHER14 (NIH3T3 cells overexpressing EGFR), and observed that in these cells too, exposure to as low as 5 μM BVD-523 for 2 h resulted in a significant elevation Erks’ phosphorylation (Fig. 10B).

Combining our observations in the hepatic-Erk1^R84H^ mice with the observations in Erk1^R84H^- and Erk1^R84S^-transformed cells and also with those in cancerous cell lines, it seems that, at least in some cancers, a low, barely detectable, Erk activity is associated with the transformed phenotype. In all cases tested here this minimal level is maintained by a robust negative feedback inhibition.

### Erk1^R84H^ imposed significant changes on the level and repertoire of NIH3T3 proteome, with Upp1 being the most elevated protein

**Fig. 11:**
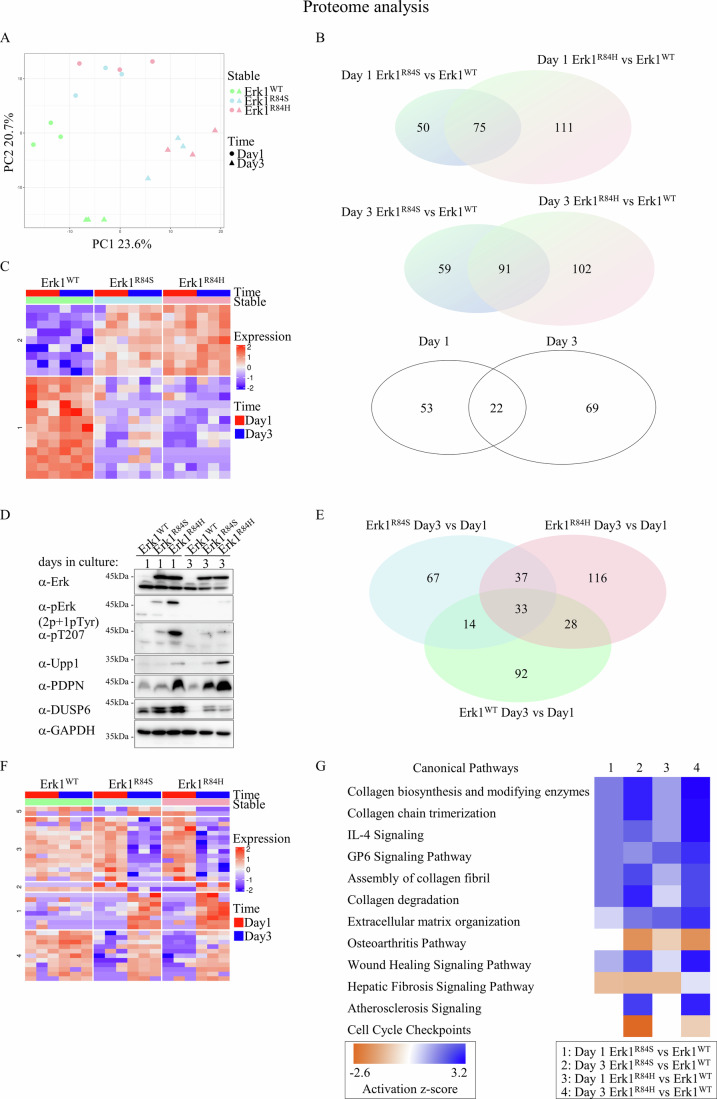
Proteomic analysis reveals distinct signatures of protein expression in NIH3T3 cells transformed by Erk1^R84S^ and Erk1^R84H^. **A** Principal component analysis (PCA) of proteomes from cells stably expressing Erk1^WT^, Erk1^R84S^, or Erk1^R84H^ at day 1 and day 3 post plating of frozen cells. PCA represents 4210 proteins with valid measurements. Note that proteome was performed on 3 independent samples of each cell line of each timepoint. **B** Venn diagrams of hierarchal pairwise comparisons of proteomic data, aimed at identification of proteins specifically expressed in Erk1^R84S^- and Erk1^R84H^-transformed cells (and not in Erk1^WT^-expressing cells). Upper pairwise comparison: of 125 proteins that are expressed in day 1 in cells transforms by Erk1^R84S^ (and not in cells expressing Erk1^WT^) and of 186 proteins expressed in day 1 in cells transforms by Erk1^R84H^ (and not in cells expressing Erk1^WT^), 75 are common. Middle pairwise comparison: of 150 proteins that are expressed in day 3 in cells transforms by Erk1^R84S^ (and not in cells expressing Erk1^WT^) and of 193 proteins expressed in day 1 in cells transforms by Erk1^R84H^ (and not in cells expressing Erk1^WT^), 91 are common. Lower pairwise comparison: of the 75 proteins specifically expressed in Erk1^R84S^- and Erk1^R84H^-transformed cells (and not in Erk1^WT^-expressing cells) in day 1 and of the 91 proteins specifically expressed in these cells in day 3, 22 are common. **C** Bioinformatic analysis revealed two major clusters of the 22 proteins specific to Erk1^R84S^- and Erk1^R84H^-transformed cells (and not in Erk1^WT^-expressing cells), downregulated and upregulated (upper and lower clusters in the heatmap, that shows all 22 proteins in all tripilicates). **D** Validation by western blot of expression levels of Upp1, DUSP6, and PDPN, discovered by the proteomic study as upregulated in Erk1^R84S^- and Erk1^R84H^-transformed cells. **E** A Venn diagram of hierarchal pairwise comparisons of proteomic data, aimed at identification of proteins differentially expressed in day 3 compared to day 1 after plating of frozen cells. In cells expressing Erk1^R84S^ 151 proteins are differentially expressed and 214 in cells expressing Erk1^R84H^. 70 of them are common. 167 proteins are differentially expressed between day 1 and 3 in cells expressing Erk1^WT^ 33 of them overlap with proteins differentially expression in both Erk1^R84S^- and Erk1^R84H^-transformed cells. Thus, 37 proteins showed altered expression between the two time points specifically in Erk1^R84S^ and Erk1^R84H^ and not in Erk1^WT^. **F** Bioinformatic analysis divided the 37 proteins identified in panel E to 5 clusters. **G** Heat map representing the z-score from pathway activated analysis (orange and blue rectangles represent activation and suppression, respectively) using Ingenuity Pathway Analysis (IPA).

As Erk1^R84H^ and Erk1^R84S^ are oncoproteins that have as yet to be explored, we sought a deeper insight into the mechanisms underlying their oncogenicity, and their efficient self-inactivation. For that we characterized the proteome and phosphoproteome of stable clones expressing Erk1^WT^, Erk1^R84S^, or Erk1^R84H^. As described, in these cells, Erk phosphorylation is commonly shut-off in these cells, but is elevated in a short time-window, within the first 24 h after thawing of frozen cultures (Fig. 7B), pointing at important differences in the biochemical wiring between this and later timepoints. Cells for proteome and phosphoroteome analysis were therefore collected 1 and 3 days post plating. This approach was justified by the proteome results. Comparing the proteomes of day 1 and day 3 revealed significant differences as reflected by the principle component analysis (PCA) (Fig. 11A; compare circle to triangles). Also, it was decided to determine the proteome of cells transformed by Erk1^R84S^ and by Erk1^R84H^ in order to increase the odds of identifying proteins specifically associated with the oncogenic process (namely, those similarly modified in the two cultures) and eliminate mutation-specific effects. Altogether we obtained the proteome and phosphoproteome (in triplicates) of cells expressing Erk1^WT^, Erk1^R84S^, or Erk1^R84H^ grown for 1 day or 3 days after plating (see PCA in Fig. 11A).

Protein lysates were analyzed via liquid-chromatography-tandem-mass-spectrometry (LC- MS/MS). The entire dataset of proteins and phosphorylation sites detected is provided in Supplementary Files S3 and S4 respectively.

The PCA calculation further shows that the proteomes of cells expressing Erk1^R84S^ or Erk1^R84H^ are remarkably similar, and significantly diverged from that of cells expressing Erk1^WT^ (Fig. 11A, blue and pink compared to green). This suggests the presence of a unique proteomic signature associated with the expression of Erk1 mutants.

Proteomic data were analyzed via several rounds of pairwise comparisons in a hierarchical manner (Fig. 11B, E) in order to accurately identify differentially expressed (DE) proteins.

To identify activities associated with the transformed phenotype, we looked for proteins elevated or decreased in clones transformed by Erk1^R84S^ or Erk1^R84H^, compared to clones expressing Erk1^WT^. 75 proteins were differentially expressed in cells expressing Erk1^R84S^ or Erk1^R84H^ on day 1 compared to cells expressing Erk1^WT^ (Fig. 11B). On day 3, this comparison revealed 91 proteins (Fig. 11B). 22 proteins were common within the two analyses and were considered specific to the transformed cells (Fig. 11B, C and Table 3).

**Table 3:** List of proteins differentially expressed in cells transformed by Erk1^R84S^ and Erk1^R84H^ to cells expressing Erk1^WT^.

Protein ID	Gene name	Day1 Erk1^R84S^ vs Erk1^WT^		Day3 Erk1^R84S^ vs Erk1^WT^		Day1 Erk1^R84H^ vs Erk1^WT^		Day3 Erk1^R84H^ vs Erk1^WT^	
		FC	*P*-value	FC	*P*-value	FC	*P*-value	FC	P-value
P52624	Upp1	22	0.00192	34	0.000684	38	0.000524	92	7.58E-05
Q62011	Pdpn	4.4	0.016371	6.7	0.003791	19	0.000107	13	0.000429
Q91Z46	Dusp7	9.2	0.000661	10	0.000474	11	0.000398	13	0.000212
Q8BLR2	Cpne4	7	0.015018	4.9	0.03802	9.6	0.006161	10	0.005503
P52927	Hmga2	3.4	0.000307	3.4	0.000319	4.2	6.96E-05	5.2	1.72E−05
Q9R002	Ifi202	2.2	0.000239	2.6	3.76E−05	3.3	4E−06	4.6	2.57E−07
P10923	Spp1	2.5	0.007333	4	0.000328	3.2	0.001489	5.6	4.38E−05
Q9WVB0	Rbpms	2.1	0.000113	2.7	7.3E−06	2.5	1.41E−05	3.2	1.49E−06
P47226	Tes	2.1	0.003046	2.1	0.002926	2.2	0.001811	2.2	0.001527
P11087	Col1a1	−2.1	0.014772	−2.4	0.004707	−2.1	0.012926	−2.9	0.001178
P24472	Gsta4	−2.8	3.58E−06	−2.6	7.84E−06	−2.6	7.82E−06	−2.4	1.99E−05
Q922W5	Pycr1	−3.1	0.003122	−3.1	0.003758	−2.9	0.005092	−2.5	0.012352
Q9D154	Serpinb1a	−2.8	0.000462	−2.6	0.000828	−3.6	7.19E−05	−3.5	8.83E−05
Q61245	Col11a1	−3.8	0.005903	−8.3	0.000179	−4.1	0.004059	−9.1	0.000119
P08074	Cbr2	−2.6	0.011436	−2.3	0.019242	−4.3	0.000513	−3.4	0.002205
P11152	Lpl	−3.1	0.000238	−4.4	1.97E−05	−4.8	9.84E−06	−6.5	1.52E−06
P18581	Slc7a2	−4.4	0.000188	−3.4	0.000901	−7.9	6.74E−06	−4.5	0.000151
Q3UTJ2	Sorbs2	−12	0.014471	−22	0.003946	−12	0.014471	−22	0.003946
Q03173	Enah	−33	1.33E−05	−4.7	0.010449	−33	1.33E−05	−27	2.63E−05
Q8K4G5	Ablim1	−12	0.007598	−8	0.019907	−43	0.000352	−74	0.000105
Q91WG0	Ces2c	−44	5.77E−05	−17	0.000731	−44	5.77E−05	−50	4.27E−05
A2ARI4	Lgr4	−18	0.000149	−3.4	0.041772	−46	9.67E−06	−7.5	0.002833

*FC* Fold change.

Uridine phosphorylase 1 (Upp1) is the most highly upregulated protein among these 22 proteins. This was validated by western blot analysis, which showed higher expression levels of Upp1 in clones expressing Erk1^R84S^ and Erk1^R84H^ compared to clones expressing Erk1^WT^ (Fig. 11D). Upp1 catalyzes the reversible phosphorolysis of uridine and deoxyuridine to uracil and ribose-1-phosphate or deoxyribose-1-phosphate. This enzyme is important for maintaining the nucleotide pool balance within cells, which is crucial for DNA and RNA synthesis and repair. However, Upp1 was proposed to fulfill a different role in cancer, related to energy metabolism. Specifically, under glucose-restricted conditions, pancreatic cancer cells were found to rely on uridine-derived ribose to fuel their metabolism. This metabolic shift is facilitated by Upp1 and is connected to Erk pathway activation, demonstrating a direct link between ERK signaling and Upp1 expression [60, 61]. It seems that a similar event occurs in the Erk1^R84S^- and Erk1^R84H^-transformed cells.

We also validated the upregulated expression of podoplanin (PDPN) (Table 3; Fig. 11D). PDPN is a transmembrane receptor glycoprotein which is used as a diagnostic marker in several types of cancer, including glioma, squamous cell carcinoma, mesothelioma, and melanoma [62]. As seen in Fig. 11D, western blot analysis confirmed the proteome analysis and revealed high expression of the protein in stable clones expressing Erk1^R84S^ or Erk1^R84H^ relative to expression level in those expressing Erk1^WT^.

We also examined other proteins that were differentially expressed (DE) in Erk1^R84S^ and Erk1^R84H^ stable clones only on either day 1 or on day 3 and did not show changes in clones expressing Erk1^WT^ (Day 1 Erk1^R84S^ vs Erk1^WT^, Day 1 Erk1^R84H^ vs Erk1^WT^, Day 3 Erk1^R84S^ vs Erk1^WT^ or Day 3 Erk1^R84H^ vs Erk1^WT^). One of these proteins is DUSP6, a phosphatase that dephosphorylates Erk1/2 and known to be involved in cell growth and differentiation [63]. On day 3, levels of DUSP6 were not monitored in cells expressing Erk1WT and were clearly measured in cells expressing Erk1^R84S^ and Erk1^R84H^. On day 1, levels were high in all clones, but higher in transformed cells, as was also validated by western blot (Fig. 11D). This observation may explain at least part of the decrease in Erk1^R84S^ and Erk1^R84H^ phosphorylation between day 1 and day 3 (Fig. 7).

Pursuing additional processes involved in the decrease in the phosphorylation of Erk1^WT^, Erk1^R84S^ and Erk1^R84H^ between day 1 and day 3, we performed the pairwise comparison presented in Fig. 11E. This analyses filters proteins whose expression is modified in day 3 compared to day 1 in each clone (Fig. 11E). Expression of 33 proteins were commonly modified between day 3 and day 1 in all three clones (Fig. 11E) and could potentially be integral to the regulation of Erk1, irrespective of the genetic background. Approximately 54% of these 33 proteins are linked to cell cycle pathways (Table 4).

**Table 4:** List of proteins whose expression is modified between day 3 and day 1 in all three clones.

Protein ID	Gene name	Erk1^WT^ Day3 vs Day1		Erk1^R84S^ Day3 vs Day1		Erk1^R84H^ Day3 vs Day1	
		FC	*P*-value	FC	*P*-value	FC	*P*-value
P53564	**Cux1**	31	0.036616	24	0.049234	25	0.048399
P53798	Fdft1	12	0.002416	5.4	0.021117	12	0.002433
Q3UUQ7	Pgap1	11	0.002461	4.1	0.042109	4.4	0.033559
Q9D273	Mmab	7.4	0.015834	7.4	0.015618	19	0.001533
Q9QZA0	Ca5b	6.7	0.019143	4.7	0.048639	17	0.001803
Q61823	**Pdcd4**	6.7	0.003423	34	2.6E−05	28	4.5E−05
P70261	Pald1	6.2	0.02215	6.2	0.021953	6.2	0.021758
O55187	Cbx4	5.4	0.015119	9.2	0.003074	5.8	0.012523
Q8BZ36	**Rint1**	5.4	0.034775	12	0.004503	−5.1	0.040165
Q9D4H9	Phf14	5.2	0.032781	7.1	0.014326	6	0.02229
Q01721	**Gas1**	5	0.011892	17	0.000254	47	1.91E−05
A8Y5H7	Sec14l1	3.4	0.000232	2.3	0.003681	2.7	0.001081
Q9DBV4	Mxra8	2.4	0.000181	2.2	0.000468	3	2.6E−05
Q9Z207	**Diaph3**	−2.1	0.000663	−2.3	0.000289	−2.3	0.000343
P32261	**Serpinc1**	−2.1	0.000165	−3.7	9.91E−07	−6	4.18E−08
Q9D0F1	**Ndc80**	−2.3	0.003187	−2.3	0.003335	−2	0.00876
Q60847	Col12a1	−2.6	0.046771	−13	7.51E−05	−14	5.78E−05
Q99JI1	Mustn1	−2.7	2.76E−05	−4.6	3.65E−07	−2.5	5.03E−05
Q9WU62	**Incenp**	−2.9	1.45E−05	−2.1	0.000355	−2.7	3E−05
P51943	**Ccna2**	−3	1.36E−06	−3.1	1.04E−06	−2.6	5.82E−06
Q00547	**Hmmr**	−3.3	0.000152	−2.3	0.002286	−2.4	0.001416
O70201	**Birc5**	−3.5	9.53E−05	−2.4	0.001436	−2.4	0.001629
P43883	Plin2	−3.6	6.78E−07	−2.9	4.61E−06	−3.6	6.68E−07
P37804	**Tagln**	−3.8	2.32E−05	−5.7	1.9E−06	−5.5	2.23E−06
Q9D1C1	**Ube2c**	−4.1	1.21E−08	−2.3	2.32E−06	−2.3	2.76E−06
Q9JJ66	**Cdc20**	−5.1	1.84E−05	−2.8	0.000997	−3.2	0.00036
Q9ERH4	**Nusap1**	−5.4	2.43E−06	−2.3	0.001214	−2.8	0.000223
P04184	Tk1	−5.8	1.82E−05	−2	0.016276	−2.3	0.006884
Q5I2A0	Serpina3g	−7	0.033585	−51	0.000483	−29	0.001552
P97477	**Aurka**	−7.6	2.7E−05	−2.2	0.022937	−2.6	0.007531
Q8R080	**Gtse1**	−9.7	2.55E−07	−3.1	0.000164	−5.2	5.95E−06
Q8BHE0	**Prr11**	−14	2.46E−06	−8.6	1.96E−05	−22	5.51E−07
Q9Z1T2	Thbs4	−16	0.001271	−63	5.27E−05	−45	0.000112

Proteins associated with cell cycle regulation are highlighted in bold.

*FC* Fold change.

37 proteins are expressed differently in day 3 compared to day 1 only in clones expressing Erk1^R84S^ and Erk1^R84H^ (Fig. 11E, F and Table 5). Within this cohort, 21 proteins exhibited upregulation and 16 downregulation. Four of the 37 proteins behaved differently in Erk1^R84S^-expressing cells as compared to Erk1^R84H^-expressing cells (Fig. 11F, clusters 2 & 5).

**Table 5:** List of proteins differentially expressed between Day 3 and Day 1, specifically in clones expressing Erk1^R84S^ and Erk1^R84H^ (21 are upregulated and 16 are downregulated).

Protein ID	Gene name	Erk1^WT^ Day3 vs Day1		Erk1^R84S^ Day3 vs Day1		Erk1^R84H^ Day3 vs Day1	
		FC	*P*-value	FC	*P*-value	FC	*P*-value
Q9QYY8	Spast	0	1	11	0.004363	46	0.000147
Q9D1Q4	Dpm3	−1.1	0.870126	−15	0.000503	31	6.9E−05
Q8R5H6	Wasf1	−2.4	0.11687	21	0.000121	19	0.000162
Q921I2	Klhdc4	1.1	0.840921	6.8	0.015834	19	0.001139
A2ARI4	Lgr4	2.6	0.111842	14	0.00065	16	0.000412
Q80ZK9	Wdtc1	−2	0.290544	28	0.000249	13	0.001842
Q9D0K1	Pex13	1.1	0.819012	4.1	0.03474	13	0.001137
Q9CWP8	Pold4	3.3	0.107246	7.8	0.012328	7.4	0.014237
P23927	Cryab	−1.4	0.360661	3.2	0.005821	6.6	0.000174
B0V2N1	Ptprs	4.1	0.095185	6.2	0.037816	5.8	0.044319
Q60793	Klf4	1.7	0.377421	48	3.58E−05	5.5	0.013268
P40338	Vhl	0	1	−5.9	0.014036	5.4	0.018417
Q78YY6	Dnajc15	3.1	0.135573	18	0.001673	5.1	0.039204
Q8C3S2	Tango6	1	0.974895	8.1	0.005513	5.1	0.021054
P58462	Foxp1	1.6	0.451002	5.8	0.011907	4.1	0.034257
Q6ZPR6	Ibtk	1.3	0.045862	2.1	9.25E−05	3.5	7.26E−07
Q9WVH9	Fbln5	0	1	3.1	0.048228	3.3	0.038753
P18581	Slc7a2	1.8	0.089191	2.3	0.021776	3.1	0.003707
Q9Z0M5	Lipa	1.8	0.004476	2.6	8.37E−05	2.7	6.45E−05
Q9CRA4	Msmo1	1.8	0.001523	2.1	0.00021	2.6	2.63E−05
Q8BGT5	Gpt2	1.4	0.162054	2	0.010143	2.2	0.00578
P10630	Eif4a2	−1.8	0.033419	−2.2	0.005843	−2.1	0.010275
P01942	Hba	1.1	0.542502	−2.2	0.00049	−2.4	0.00026
Q91YK2	Rrp1b	−1.1	0.771838	−2.5	0.012502	−3	0.004568
Q9CQ19	Myl9	−1.3	0.054072	−2.5	1.13E−05	−3	1.54E−06
E9Q414	Apob	−1.1	0.757226	−2.7	0.000272	−3	9.93E−05
Q61838	A2m	−1.2	0.309238	−3.1	1.51E−05	−4.4	1.13E−06
Q3V0B4	Ccdc108	−1.2	0.317086	−3.6	4.49E−06	−5.1	3.67E−07
Q9R0G6	Comp	−1.5	0.527252	−23	0.000682	−5.4	0.028499
Q8VDG6	Mlk4	1.1	0.880009	−5.1	0.016009	−6.7	0.00666
Q8CHT3	Ints5	2.3	0.298635	−12	0.008757	−7.5	0.025675
Q3UGP9	Lrrc58	1.8	0.489965	−7.8	0.032309	−7.9	0.031323
Q8R0W0	Eppk1	2.7	0.119767	12	0.001382	−9.2	0.002986
P60824	Cirbp	−1	0.973381	13	0.001047	−12	0.001281
Q9ES30	C1qtnf3	−1.2	0.795499	−47	8.79E−05	−18	0.000857
P62737;P63268	Acta2;Actg2	−1.5	0.478371	−7.7	0.002748	−22	0.000116
Q64096	Mcf2l	−1.4	0.703606	−40	0.000933	−67	0.000352

*FC* Fold change.

Among the upregulated proteins, Spast stands out when comparing day 3 to day 1 in Erk1^R84H^ stable clones. It was also seen to be upregulated when comparing day 3 to day 1 in Erk1^R84S^ stable clones but to a lesser extent (Table 5). Spast, also known as spastin, is involved in microtubule severing and is crucial for cytoskeletal dynamics and intracellular transport, which can influence cell proliferation and migration [64]. The upregulation of Spast suggests alterations in cytoskeletal regulation and cellular dynamics in Erk1^R84H^-expressing cells. Another protein that was enriched in cells expressing Erk1^R84S^ and Erk1^R84H^ mutants when comparing day 3 to day 1, is PTPRS, a receptor-type protein tyrosine phosphatase (PTP). PTPRS has been reported to negatively regulate the RAS pathway in colorectal cancer (CRC) by dephosphorylating Erk, leading to its deactivation and preventing its nuclear translocation [65]. This suggests that PTPRS could play an important role in the observed decrease in Erk phosphorylation in the Erk1^R84S^ and Erk1^R84H^ mutants.

Among the proteins that were downregulated in the comparison between day 3 and day 1 in cells expressing the active Erks is MCF2L, a Guanine nucleotide exchange factor (GEF) that activates RHOA and CDC42 [66]. The depletion of MCF2L expression in the mutants suggests a potential impairment in processes such as cytoskeletal dynamics, cell migration, and proliferation. Together with the upregulation of Spast it is apparent that these proteins play a part in the process of oncogenic transformation Erk1^R84S^ and Erk1^R84H^, by altering the cytoskeleton.

Proteomic data were further analyzed via Ingenuity Pathway Analysis (IPA) (Fig. 11G). This analysis showed that pathways associated with collagen synthesis, such as “Collagen biosynthesis and modifying enzymes,” “Collagen chain trimerization,” and “Collagen degradation,” were consistently downregulated in cells transformed by Erk1^R84S^ or Erk1^R84H^ (Fig. 11G). Collagen expression in cancer varies, and, depending on the type of cancer, both increase and decrease can be important. The downregulation of both collagen synthesis and degradation in our model suggest an imbalance in the dynamics of extracellular matrix. In contrast, pathways that are closely linked to tumorigenesis, such as “Cell Cycle Checkpoints” were significantly upregulated in the transformed cells (Fig. 11G).

In summary, the comprehensive proteomic analysis reveals a profile that is consistent with neoplastic processes and tumorigenesis (Fig. 11). Specifically, Erk1^R84S^ and Erk1^R84H^ rewire cellular energy metabolism, cytoskeleton organization and cell cycle machinery. With respect to the negative feedback loop of their own phosphorylation, Erk1^R84S^ and Erk1^R84H^ upregulate the expression levels of DUSP6, DUSP7 and PTPRS. We hypothesize that these phosphatases play a crucial role in mediating the feedback mechanism responsible for the observed suppression of Erk phosphorylation.

### Phosphoproteomes of cells transformed by Erk1^R84H^ or Erk1^R84S^ reveal high activity of kinases associated with cell cycle and apoptosis, as well as with the mTOR pathway, and low activity of kinases involved in DNA damage response

The complete phosphoproteome dataset is provided in Supplementary File S4. Hundreds of phosphorylation sites were identified as being differentially phosphorylated in cells transformed by Erk1^R84S^ and Erk1^R84H^. However, for most of these sites, the kinases responsible for their phosphorylation, as well as the functional consequences of these modifications, are still unreported. Below we describe a bioinformatic analysis of sites for which information is available.

#### A distinct phosphoproteome signature common to Erk1^R84S^ and Erk1^R84H^

**Fig. 12:**
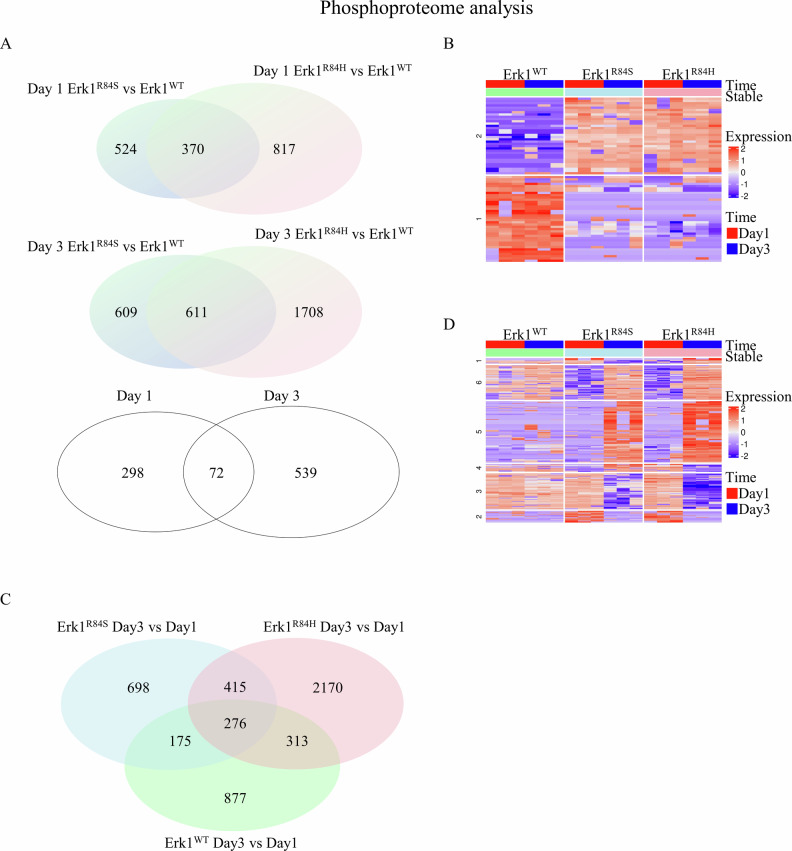
Distinct phosphorylation signatures in NIH3T3 cells expressing oncogenic Erk1^R84S^ and Erk1^R84H^ revealed by Phosphoproteomic analysis. **A** Venn diagrams of pairwise comparisons of phosphor-proteomic data aimed at identification of phosphorylation sites modified specifically in NIH3T3 cells expressing Erk1^R84S^ or Erk1^R84H^, compared to cells expressing Erk1^WT^. Upper diagram: of 894 phosphorylation sites modified in day 1 in Erk1^R84S^-transformed cells compared to cells expressing Erk1^WT^ and of 1187 sites modified in cells transformed by Erk1^R84H^, 370 are common. Middle diagram: of 1220 sites specifically modified in day 3 in cells expressing Erk1^R84S^ and of 2319 sites modified in cells expressing Erk1^R84H^, 611 are common. Lower diagram: of the 370 sites modified in day 1 specifically in clones expressing Erk1^R84S^ and Erk1^R84H^ and of the 611 sites specifically modified in day 3, 72 are common. **B** Heatmap of the 72 shared phospho-sites in cells expressing Erk1^R84S^ or Erk1^R84H^ on both days but absent in Erk1^WT^, divided to 2 clusters of upregulated and downregulated phosphorylation in cells expressing Erk1^R84S^ or Erk1^R84H^ compared to cells expressing Erk1^WT^. **C** Venn diagram of hierarchal pairwise comparisons of phosphor-proteomic data aimed at identification of differentially phosphorylated phosphosites between day 1 and day 3 of the culture’s life. Of 1546 sites differentially phosphorylated between day 3 and day 1 in cells expressing Erk1^R84S^ and of 3174 sites differentially phosphorylated in cells expressing Erk1^R84H^, 691 are common. Of 1328 sites modified between day 3 and day1 in cells expressing Erk1^WT^, 276 are common with sites modified in cells expressing Erk1^R84S^ or Erk1^R84H^. Thus, 415 phosphoacceptors are differentially phosphorylated on day 3 vs day 1 in cells transformed by Erk1^R84S^ or Erk1^R84H^ and not in cells expressing Erk1^WT^. **D** Heatmap of the 415 phospho-sites differentially phosphorylated on day 3 vs day 1 in the transformed cells, divided into six clusters as revealed by bioinformatics analysis.

When analyzing the phospho-proteomics data, we first looked for phospho-acceptors that were differentially phosphorylated in clones expressing Erk1^R84S^ or Erk1^R84H^ as compared to clones expressing Erk1^WT^. These comparisons revealed 370 and 611 phosphorylation sites on day 1 and day 3, respectively (Fig. 12A, B). 72 of these were common in day 3 and day 1 (Table 6) and are considered specific to the transformed cells.

**Table 6:** Phosphorylation sites differentially phosphorylated on both Day 1 and Day 3 in clones expressing Erk1^R84S^ or Erk1^R84H^ compared to Erk1^WT^.

Protein ID	Residue number	Gene name	Day1 Erk1^R84S^ vs Erk1^WT^		Day3 Erk1^R84S^ vs Erk1^WT^		Day1 Erk1^R84H^ vs Erk1^WT^		Day3 Erk1^R84H^ vs Erk1^WT^	
			FC	*P*-value	FC	*P*-value	FC	*P*-value	FC	P-value
P58871	1028	Tnks1bp1	760	0.0018	54	0.03482	720	0.0019	92	0.01946
Q99NB9	488	Sf3b1	620	4.6E−05	−43	0.00385	350	0.00011	−43	0.00385
Q9CQS8	13	Sec61b	380	0.00022	71	0.00301	50	0.00535	56	0.00454
Q99P72	98	Rtn4	320	3.9E−05	200	9E−05	1000	6.1E−06	26	0.0045
Q6DID5	105	Mum1	270	0.0013	23	0.04057	41	0.01839	170	0.00245
Q9Z2H5	666	Epb41l1	230	0.00014	32	0.00455	250	0.00011	240	0.00012
P10923	61	Spp1	230	0.00322	49	0.02291	54	0.02021	73	0.01388
P10923	62	Spp1	230	0.00322	49	0.02291	54	0.02021	73	0.01388
P52480	41	Pkm	220	1.5E−05	16	0.00462	460	3.9E−06	51	0.00029
Q68FD5	105	Cltc	200	3.4E−11	150	7.3E−11	210	3E−11	200	3.4E−11
P27361	207	Mapk3	180	0.00913	87	0.02118	590	0.00244	360	0.00428
P97855	231	G3bp1	160	0.00098	49	0.00651	28	0.01591	58	0.00501
P97855	230	G3bp1	160	0.00098	49	0.00651	28	0.01591	58	0.00501
P97855	229	G3bp1	160	0.00289	180	0.00251	28	0.0323	37	0.02199
Q3UVY1	572	Gpr149	74	0.00408	62	0.00535	27	0.01955	17	0.04087
P22518	139	Clk1	68	0.02042	390	0.00254	70	0.01957	61	0.02331
Q6PEE2	18	Ctif	58	0.02343	78	0.01663	660	0.00126	120	0.01005
D3YXK2	366	Safb2	56	7.4E−05	240	3.1E−06	44	0.00013	210	4E−06
D3YXK2	372	Safb2	56	7.4E−05	240	3.1E−06	44	0.00013	210	4E−06
P17182	254	Eno1	53	0.00692	34	0.01357	74	0.00409	370	0.00036
Q9JIK5	387	Ddx21	51	0.00337	23	0.01424	18	0.02174	24	0.01294
Q9CQS8	19	Sec61b	47	0.03061	71	0.01837	50	0.028	56	0.02484
Q3UVY1	517	Gpr149	41	0.00566	19	0.02129	47	0.00449	200	0.00041
P70398	2547	Usp9x	37	0.01272	50	0.00815	63	0.00581	95	0.00309
Q99MD9	648	Nasp	33	0.01824	130	0.00239	19	0.0424	110	0.00308
O55003	85	Bnip3	30	0.00568	290	0.0001	250	0.00013	450	5E−05
Q9D7X1	20	Kctd4	28	0.0047	180	0.00015	200	0.00012	460	2.9E−05
Q8VBV3	124	Exosc2	16	0.03451	26	0.01644	18	0.02995	26	0.01642
Q60605	57	Myl6	8.9	1.8E−05	6.2	0.00011	7	5.6E−05	7	5.8E−05
Q8CE50	40	Snx30	4.3	0.00029	2.3	0.01291	3.9	0.00053	2.7	0.0055
P52927	100	Hmga2	2.9	0.03303	2.9	0.0365	2.7	0.04588	3.8	0.01079
P52927	104	Hmga2	2.9	0.03303	2.9	0.0365	2.7	0.04588	3.8	0.01079
P52927	104	Hmga2	2.6	0.00934	2.6	0.00992	3.8	0.00098	4.5	0.00035
P52927	44	Hmga2	2.3	0.04222	4.1	0.0025	3.3	0.00761	5.9	0.00041
Q9ET54	901	Palld	−2.2	0.01929	−4.8	0.00015	−3.3	0.00166	−9	5.3E−06
Q8K4G5	738	Ablim1	−2.2	0.0203	−2.8	0.00551	−3.8	0.00078	−5.4	9.3E−05
Q8VDP3	777	Mical1	−2.2	0.02564	−2.2	0.02415	−3.1	0.00302	−2.3	0.0216
Q8K4G5	789	Ablim1	−2.4	0.01949	−2.5	0.01312	−5.1	0.00024	−4.7	0.00038
Q01279	695	Egfr	−2.5	0.03173	−5.4	0.00081	−6.9	0.00025	−8.2	0.00011
Q8BRV5	205	Kiaa1671	−2.7	0.00543	−2.2	0.01989	−3.3	0.00134	−2.7	0.00451
Q8K4G5	475	Ablim1	−2.9	0.02284	−3	0.02102	−7.7	0.00029	−8.4	0.0002
O55098	950	Stk10	−2.9	0.00285	−2.9	0.00278	−3.9	0.00047	−4.2	0.00028
P63085	183	Mapk1	−10	0.03017	−370	3.3E−05	−11	0.02407	−1600	3.3E−06
P63085	185	Mapk1	−10	0.03017	−370	3.3E−05	−11	0.02407	−1600	3.3E−06
Q8C4S8	497	Dennd2a	−13	0.0294	−14	0.02476	−13	0.0294	−14	0.02476
Q6DFW0	9		−14	0.02773	−28	0.00812	−14	0.02773	−110	0.00075
Q9R1E0	316	Foxo1	−14	0.02983	−16	0.02557	−14	0.02983	−16	0.02557
P51827	908	Aff3	−18	0.04494	−36	0.01601	−18	0.04494	−36	0.01601
Q69ZW3	719	Ehbp1	−19	0.02342	−78	0.00212	−19	0.02342	−19	0.02173
P30549	341	Tacr2	−22	0.0056	−54	0.00093	−22	0.0056	−54	0.00093
Q99MI1	824	Erc1	−22	0.01711	−43	0.00563	−22	0.01711	−160	0.00067
Q8BI84	1915	Mia3	−23	0.0249	−22	0.0272	−23	0.0249	−22	0.0272
Q8K019	275	Bclaf1	−25	0.03886	−29	0.03163	−140	0.00378	−170	0.00271
P30549	345	Tacr2	−26	0.00484	−54	0.00115	−26	0.00484	−54	0.00115
Q8K4G5	411	Ablim1	−32	0.00093	−160	3E−05	−130	4.6E−05	−160	3E−05
Q80ZK9	513	Wdtc1	−32	0.02052	−23	0.03344	−32	0.02052	−23	0.03344
P25799	452	Nfkb1	−33	0.03057	−200	0.0028	−33	0.03057	−31	0.03329
Q9WTQ5	234	Akap12	−42	0.04107	−53	0.03161	−55	0.03037	−320	0.00397
Q4KMM3	202	Oxr1	−57	0.00371	−440	0.00014	−57	0.00371	−440	0.00014
Q8R1A4	1428	Dock7	−63	0.0036	−360	0.00023	−63	0.0036	−360	0.00023
P97445	2252	Cacna1a	−66	9.9E−11	−94	3.5E−11	−66	9.9E−11	−94	3.5E−11
Q8BVL3	421	Snx17	−68	0.01485	42	0.02752	−510	0.00114	50	0.02178
P23475	518	Xrcc6	−69	4.2E−10	−4.1	0.0001	−69	4.2E−10	−3.9	0.00015
Q6QI06	1176	Rictor	−76	0.00102	−21	0.01102	−19	0.01326	−66	0.00133
Q6QI06	1174	Rictor	−76	0.00102	−21	0.01102	−19	0.01326	−66	0.00133
P97445	2220	Cacna1a	−85	3.2E−11	−160	5.6E−12	−85	3.2E−11	−160	5.6E−12
O35730	324	Ring1	−86	0.0003	17	0.0084	−86	0.0003	17	0.00782
Q91YM2	589	Arhgap35	−87	0.00108	−33	0.00589	−87	0.00108	−32	0.0062
Q8BJ37	132	Tdp1	−89	0.02047	−83	0.02197	−89	0.02047	−640	0.0022
P39447	1576	Tjp1	−120	0.00511	−43	0.02012	−23	0.04597	−43	0.02012
P59222	626	Scarf2	−180	6.3E−06	−89	2.7E−05	−180	6.3E−06	−27	0.00049
Q9ERU9	2342	Ranbp2	−310	0.00419	−100	0.01503	−63	0.02644	−150	0.01009

*FC* Fold change.

Among these 72 sites, to our knowledge, only 8 sites were linked to specific kinases, three of these sites are found on Erk1 itself (Thr202, Tyr204, and Thr207). As expected (Fig. 7) phosphorylation of Thr202 and Tyr204 were downregulated and that of Thr207 was upregulated in clones expressing the mutants relative to clones expressing Erk1^WT^ (verified by western blot on the same samples; Fig. 11D).

Three other sites were identified as Erk1/2 substrates: Ser366 of SAFB, Thr696 of EGFR, and Thr950 of STK10. SAFB (Scaffold Attachment Factor B) is involved in chromatin organization and transcriptional regulation. Its elevated phosphorylation could affect gene expression and chromatin structure. Phosphorylation of the Epidermal Growth Factor Receptor (EGFR) at Thr696, which was downregulated in cells expressing the Erk mutants, is known to inhibit EGFR’s signaling capacity. STK10 (Serine/Threonine Kinase 10) is involved in the regulation of lymphocyte signaling and cytoskeletal dynamics. The remaining two phosphorylation sites are Ser44 and Ser100 of High Mobility Group AT-Hook 2 (HMGA2), involved in chromatin remodeling and regulation of gene expression. HMGA2 is often associated with cancer progression and metastasis. Phosphorylation at Ser44, regulated by CDK1, and at Ser100, regulated by CSNK2A1 (CK2A1), which were downregulated in cells expressing the active Erks, probably affect its role in controlling chromatin dynamics.

We applied yet another approach for data analysis in which we analyzed phosphorylated sites whose levels were highly differentiated (a fold change of >200 or <-100 in cell expressing Erk1^R84S^ Erk1^R84H^ relative to those expressing Erk1^WT^). This analysis is summarized in Tables 7 and 8. Since the exact downstream effects of these phosphorylation events have not been fully investigated, several key biological processes, summarized in Tables 7 and 8, can be inferred from the known functions of the proteins involved.

**Table 7:** Phosphorylation sites enriched in cells expressing Erk1^R84S^ and Erk1^R84H^ compared to Erk1^WT^.

Gene name	Residue	Day1 Erk1^R84S^ vs Erk1^WT^		Day3 Erk1^R84S^ vs Erk1^WT^		Day1 Erk1^R84H^ vs Erk1^WT^		Day3 Erk1^R84H^ vs Erk1^WT^		Biogical process
		FC	*p*val	FC	*p*val	FC	*p*val	FC	*p*val	
Rtn4	98	320	3.9E−05	200	9.02E−05	1000	6.08E−06	26	0.004503	Apoptosis and autophagy
Tnks1bp1	1028	760	0.001802	54	0.034819	720	0.0019	92	0.019463	Immune response and inflammation
Ctif	18	58	0.02343	78	0.016634	660	0.00126	120	0.010045	Cell cycle regulation and proliferation
MAPK3	207	180	0.009134	87	0.021177	590	0.002442	360	0.004276	Cell cycle regulation and proliferation
Pkm	41	220	1.52E−05	16	0.004618	460	3.88E−06	51	0.000295	Metabolic reprogramming
Sf3b1	488	620	4.57E−05	−43	0.003847	350	0.00011	−43	0.003847	RNA processing and splicing
Bnip3	85	30	0.00568	290	0.000101	250	0.000128	450	5.02E−05	Apoptosis and autophagy
Epb41l1	666	230	0.000137	32	0.004552	250	0.000114	240	0.000122	Cytoskeletal dynamics and cell adhesion
Cltc	105	200	3.42E−11	150	7.27E−11	210	2.96E−11	200	3.38E−11	Cytoskeletal dynamics and cell adhesion
Kctd4	20	28	0.004702	180	0.000145	200	0.000124	460	2.94E−05	Protein transport and signal transduction
Sec61b	13	380	0.000222	71	0.003009	50	0.005346	56	0.004536	Protein transport and signal transduction
Mum1	105	270	0.001302	23	0.040567	41	0.018391	170	0.00245	Chromatin remodeling and transcriptional regulation
Spp1	61	230	0.003225	49	0.022909	54	0.02021	73	0.013878	Immune response and inflammation
Spp1	62	230	0.003225	49	0.022909	54	0.02021	73	0.013878	Immune response and inflammation
Eno1	254	53	0.006923	34	0.013573	74	0.004087	370	0.000361	Metabolic reprogramming
Safb;Safb2	366	56	7.36E−05	240	3.12E−06	44	0.00013	210	4.04E−06	RNA processing and splicing
Safb;Safb2	372	56	7.36E−05	240	3.12E−06	44	0.00013	210	4.04E−06	RNA processing and splicing
Clk1	139	68	0.020415	390	0.002535	70	0.019569	61	0.023313	Chromatin remodeling and transcriptional regulation

*FC* Fold change.

**Table 8:** Phosphorylation sites downregulated in cells expressing Erk1^R84S^ and Erk1^R84H^ compared to Erk1^WT^.

Gene name	Residue	Day1 Erk1^R84S^ vs Erk1^WT^		Day3 Erk1^R84S^ vs Erk1^WT^		Day1 Erk1^R84H^ vs Erk1^WT^		Day3 Erk1^R84H^ vs Erk1^WT^		Biological process
		FC	*p*val	FC	*p*val	FC	*p*val	FC	*p*val	
Snx17	421	−510	0.001141	−68	0.014848	50	0.021775	42	0.027525	Apoptosis and transcriptional regulation
Scarf2	636	−180	6.29E−06	−180	6.29E−06	−27	0.00049	−89	2.75E−05	Signal transduction and immune response
Bclaf1	275	−140	0.003779	−25	0.038858	−170	0.002712	−29	0.031634	Apoptosis and transcriptional regulation
Ablim1	411	−130	4.56E−05	−32	0.000929	−160	2.99E−05	−160	2.99E−05	Cytoskeletal organization and cell migration
Ranbp2	2342	−63	0.026437	−310	0.004188	−150	0.010088	−100	0.015025	Nucleocytoplasmic transport and RNA processing
Mapk1	183	−11	0.024074	−10	0.030166	−1600	3.34E−06	−370	3.31E−05	Cell cycle regulation and proliferation
Mapk1	185	−11	0.024074	−10	0.030166	−1600	3.34E−06	−370	3.31E−05	Cell cycle regulation and proliferation
Tdp1	132	−89	0.020473	−89	0.020473	−640	0.002198	−83	0.021969	DNA repair
Oxr1	202	−57	0.003714	−57	0.003714	−440	0.000143	−440	0.000143	Oxidative stress response
Dock7	1428	−63	0.003602	−63	0.003602	−360	0.000231	−360	0.000231	Cytoskeletal organization and cell migration
Akap12	234	−55	0.030365	−42	0.041067	−320	0.003974	−53	0.031607	Cytoskeletal organization and cell migration
Cacna1a	2220	−85	3.21E−11	−85	3.21E−11	−160	5.58E−12	−160	5.58E−12	Calcium ion transport and neuronal signaling
Erc1	824	−22	0.017108	−22	0.017108	−160	0.000672	−43	0.005634	Synaptic vesicle exocytosis
C9orf72	9	−14	0.027733	−14	0.027733	−110	0.000746	−28	0.008115	Nucleocytoplasmic transport and RNA processing
Nfkb1	452	−33	0.030568	−33	0.030568	−31	0.033294	−200	0.002797	Signal transduction and immune response

*FC* Fold change.

We further explored the phosphoproteomics data by conducting pairwise comparisons of phosphorylation sites between different time points within each stable cell line, specifically focusing on Erk1^R84S^ and Erk1^R84H^ mutants (Fig. 12C). In these analyses, we excluded phosphorylation sites that were also differentially phosphorylated in the Erk1^WT^ stable line, allowing us to isolate phosphorylation events unique to the active mutants. This approach identified 1113 phosphorylation sites in Erk1^R84S^ and 2585 phosphorylation sites in Erk1^R84H^, with 415 sites shared between the two mutants (Fig. 12C, D). These 415 phosphorylation sites are particularly noteworthy as they represent a distinct phosphoproteome signature associated with Erk1^R84H^ and Erk1^R84S^. Given the limited available information on the downstream effects of many of these phosphorylation sites, further investigation is required to elucidate their roles in the Erk1-mediated oncogenic process.

In any case, the phosphoproteome analysis revealed a specific phosphorylation patterns associated with Erk1^R84S^- and Erk1^R84H^-transformed cells.

We sought next to investigate possible upstream regulators, particularly to identify protein kinases whose activity differs significantly between the clones and between time points. To achieve this, we used the Kinase-substrate enrichment analysis (KSEA) tool. Tables 9 and 10 summarize the significant upregulated and downregulated kinases as predicted by KSEA.

**Table 9:** Protein kinases exhibiting differential activity between Day 3 and Day 1 across all clones.

	Higher activity in group 2	Higher activity in group 1
Group 1: Erk1^WT^ day1 Group 2: Erk1^WT^ day3	CDK1, AURKB, CDK2, HIPK2 & AURKA	CLK1, CSNK2A1, MAPK14 & CLK2
Group 1: Erk1^R84S^ day1 Group 2: Erk1^R84S^ day3	HIPK2, CDK2, AURKA, CDK1, CDC7 & CLK2	PRKAA2
Group 1: Erk1^R84H^ day1 Group 2: Erk1^R84H^ day3	CDK1, CDK2, HIPK2, AURKA, PRKACA, CAMK2A, PAK2, AKT1, MAPK8 & PRKCA	CSNK2A1, CSNK2A2 & MAPK14

**Table 10:** Protein kinases exhibiting differential activity in clones transformed by Erk1^R84H^ or Erk1^R84S^ compared to clones expressing Erk1^WT^.

	Higher activity in group 2	Higher activity in group 1
Group 1: Erk1^WT^ day1 Group 2: Erk1^R84S^ day1	MTOR	CSNK2A2, PRKDC, CLK1, CSNK2A1 & GSK3A
Group 1: Erk1^WT^ day1 Group 2: Erk1^R84H^ day1	AURKB, MTOR & CDC7	CLK2, CSNK1D & CAMK2A
Group 1: Erk1^WT^ day3 Group 2: Erk1^R84S^ day3	CLK2, CDK5, EIF2AK2,CDC7, CLK1 & MAPK14	CDK1 & GSK3B
Group 1: Erk1^WT^ day3 Group 2: Erk1^R84H^ day3	PRKCG, AKT1, MAPK8, PRKAA2, PRKAA1, MTOR, PRKACA, CDK5 & CAMK2A	CSNK2A2 & CSNK2A1

Comparing kinase activity between Day 1 and Day 3 across all three clones revealed a progression towards cell cycle entry, proliferation, and DNA replication, as evidenced by the upregulation of CDK1, AURKB, CDK2, and AURKA. Concurrently, there is an indication of increased pro-apoptotic activity reflected by the higher activity of HIPK2 (Table 9). This suggests a complex interplay between cell proliferation and apoptosis regulation, potentially indicating a balance between pro- and anti-apoptotic signaling. Comparing kinase activity between day 1 and day 3 in cells expressing Erk1^R84S^, we observed, in addition to the progression towards cell cycle entry and DNA replication, higher activity of CLK2, a kinase involved in RNA splicing and pre-mRNA processing. We also observed a decrease in the activity of PRKAA2, which is the catalytic subunit of the AMP-activated protein kinase (AMPK). This points at changes in energy metabolism and stress. A similar comparison in cells expressing Erk1^R84H^ revealed that survival signaling were upregulated (reflected, for example, by AKT1, PRKACA and PRKCA activities) and a focus on cytoskeletal reorganization, as indicated by increased activity of PAK2 and CAMK2A. We also observed a decreased activity of CSNK2A1, CSNK2A2, and MAPK14 (p38α), suggesting a suppression of both DNA repair mechanisms and inflammatory pathways (Table 9).

Notably, when comparing Erk1^R84S^ and Erk1^R84H^ clones to Erk1^WT^, the mTOR kinase was specifically upregulated in clones transformed by the active Erks on day 1 and in Erk1^R84H^ on day 3, suggesting enhanced cellular growth and supporting the notion that Erk1^R84H^ and Erk1^R84S^ modify the cells’ metabolism (Table 10). In contrast, kinases involved in DNA damage response (e.g., CSNK2A2, PRKDC) were downregulated in clones expressing either Erk1^R84H^ or Erk1^R84S^ compared to Erk1^WT^ (Table 10).

## Discussion

This study has shown that Erk1^R84H^ is a bona fide oncoprotein in vivo. The Erk MAP kinases have been proposed to be associated with cancer, but evidence to support this notion were circumstantial and correlative. Those presumptions were based on observations that Erks activity is high in some cancers and cancer models [7, 8, 16–18, 21, 67]. It was proposed in fact that oncogenic Ras and Raf promote cancer by elevating Erk activity [68, 69], a notion supported by the functions of Erk as an enhancer of the cell cycle [19, 70] and as an inhibitor of cell death [71]. Additionally, all components functioning upstream of Erks in the RTK-Ras-Raf-MEK pathway are mutated in cancer, and Erks have been shown to be required for tumor development, for example in a mouse model of non-small cell lung carcinoma [72]. These observations could not be conclusive with respect to whether Erks are the major or sole mediators of pathway’s oncogenicity because of several reasons. First, some upstream components, such as EGFR, other RTKs, and Ras, activate multiple downstream effectors, in addition to the Raf-MEK-Erk arm, including cancer promoting components (e.g., the PI3K-AKT/PKB pathway, RalGDS, PKCε, PKCζ, Src; [3, 4, 33, 35, 73, 74]. Second, in some tumors in which mutated Ras or B-Raf are the tumor drivers Erk activity is not significantly elevated [20, 40, 75, 76]. Third, Erk mutants are very rare in cancer [42].

The finding described here, that there exists an oncogenic version of Erk, suggests that Erks (perhaps primarily Erk1) are one of the most critical, if not the only, mediators of the oncogenicity of the RTK-Ras cascade. Furthermore, the dynamics of Erk1^R84H^ activity in the course of oncogenesis, i.e., its strong downregulation, suggests that also in those cases of Ras-, Raf-, or MEK-driven cancer, where Erk activity is not readily detectable, Erk may be an important driver of the malignancy.

It is not clear why Erk1^R84H^, being a potent oncoprotein, is so rarely found in human tumors. This riddle may be explained by the fact that oncogenic EGFR or Ras activate an array of tumor-promoting pathways in addition to the Erk cascade, making their oncogenicity more powerful and more resistant to cellular protective systems. B-Raf may also activate more components than MEKs. Perhaps activation of Erk alone in a given cell, with no support of other oncogenic pathways, anti-apoptotic for example, allows the mutated cell and the body systems to respond more efficiently. The very efficient negative feedback activity of the Erk1^R84H^ molecule may also contribute to shut-off of parallel pathways. In the transgenic model, in which Erk1^R84H^ is induced in all hepatocytes simultaneously, there are high chances that some cells will escape the anti-cancer responses and will give rise to tumors. This notion may explain why the number of cancer cases with mutated Ras is very high, while the number of cases with mutated B-Raf is lower (and restricted to some cancers) and those with mutated MEK is much lower, and with mutated Erk the cases are very rare. In any case, the fact that the R84H mutation in ERK1 is found in different types of cancer may suggest that the mutation is not restricted to some types of the disease, and although not reported yet in HCC we predict that it would be found in this cancer too. Accordingly, we predict that if we take advantage of our mouse model to direct expression of Erk1^R84H^ to other tissues (i.e., skin, intestine, pancreas), tumors will be developed in those organs.The observation that Erk promotes cancer, while suppressing its own activity and probably activity of other pathway’s upstream components, may argue that when Erk1 is constitutively active, even at low level, RTKs and Ras effectors are not required for tumor maintenance. Moreover, it is possible that the powerful negative feedback that maintains low activity of the pathway is crucial for survival of the tumor. High levels of Erk activity were reported to be hazardous to both normal and to even a greater extent to cancer cells, observations that brought up the “Goldilocks principle”, which suggests that a given level of activity would promote and maintain tumorigenesis [37, 77, 78]. Yet, the almost complete abolishment of Erk activity observed in the hepatic-Erk1^R84H^ mouse model, the Erk1^R84S^- and Erk1^R84H^-transformed cells and in cancer-derived cell lines suggest that for cancer development the “Goldilocks principle” does not lead to some intermediate Erk activity, but rather to a minimal activity.

It must be taken into consideration that in the living growing cell, Erk activity was reported to significantly fluctuate [79, 80]. In the cells transformed by Erk1^R84H^ or Erk1^R84S^ too, Erks were upregulated at various timepoints (Fig. 6). Thus, the low, barely detectable, activity is clearly not constant. The different levels of Erk activity in different cases and the fluctuation of the activity levels complicate therapeutic efforts. These efforts should not focus entirely on inhibitors. Although it seems counterintuitive, activation of proto-oncogenes may be considered, and should take into consideration the exact timing of treatment [37].

The mouse model generated in this study could be readily used to monitor the effect of Erk1^R84H^ in any cell type desired. Here the expression was directed to the liver and the outcome was a severe HCC. The disease evolved quite rapidly in all hepatic-homo-Erk1^R84H^ mice, although Erk1^R84H^ was not assisted by other oncogenes and the mice were not deprived of tumor suppressors. This makes the hepatic-Erk1^R84H^ mouse a useful and reliable model for studying this cancer. Such models are of importance given the increase in HCC cases. Driven by the high rate of fatty liver diseases in western societies. 30% of Americans suffer of metabolic dysfunction-associated steatotic liver disease (MASLD) 2–5% whom may develop HCC over time [81]. HCC has become the third leading cause of cancer-related mortality worldwide [82].

Several mouse models are currently used for studying HCC in mice by activating the EGFR-Ras-Raf-MEK-Erk pathway. Some utilize carcinogens, with or without high fat diets, others involve expressing viral proteins (derived from hepatitis B, or Hepatitis C viruses), or expressing genes such as Myc, astrocyte elevated gene 1, E2F1, TGFα or β-catenin, or injection of relevant proteins via hydrodynamic tail vein infection (for review on HCC preclinical models see [48, 83, 84]. These models may require repeated injections, continuous provision of high-fat diet for many months, or injection of plasmid or viruses expressing Cre recombinase.

The novel hepatic-Erk1^R84H^ mice model described here is very specific to the outcome of this pathway, as it specifically activates the downstream component of the cascade. To our knowledge, no other transgenic model for HCC directly activates this cascade. Additionally, the hepatic-Erk1R84H mice model is rather simple, and requires just provision of dox-supplemented diet.

Understanding Erk1^R84H^-induced cancer and devising treatments that target this process, are important not only with respect to primary tumors caused by Erk1^R84H^, but also of tumors driven by other oncogenes that develop resistance to inhibitors of RTKs, Rafs and MEKs. Acquiring mutations in R84 of Erk1 could be the mechanism of drug resistance in these patients, as mutations in R84 have already been identified in screens for drug-resistant cells [42, 85, 86].

Tumor development in whole animals following the induction of Erk1^R84H^ expression in the liver is clearly apparent within 5 months and disease is severe, forcing sacrifice of the animals due to extreme suffering. Also, the kidney is severely affected in the hepatic-Erk1^R84H^ mouse model, providing a convenient system for studying the liver-kidney cross talk in HCC. Acute kidney injury is frequently observed in cancer and may affect 31.8% of liver cancer patients (based on study with Danish cancer patients; [87–89]). Liver-kidney crosstalk is complex, involving many system components and it is not clear how these relationships are affected in HCC [90].

Upp1, here reported to be prominently upregulated in cells expressing activated Erk, has recently been reported to be associated with cancer development as an integral component of the metabolic adaptation machinery of the tumor, required for maintaining the transformed phenotype [60, 61]. These findings underscore the importance of metabolic pathways in neoplastic transformation and highlight Upp1 as a potential target for therapeutic intervention in Erk1-driven cancers.

Feedback pathways within the Erk pathway have been reported to involve activation of phosphatases, as well as phosphorylation of upstream components at inhibitory phospho-sites, catalyzed by downstream molecules, mainly Erks [57–59, 91–94] (Reviewed in refs. [2] and [54]).

It was also proposed that interfering with the resultant cascade is not necessarily therapeutic, as inhibitors may disturb the negative feedback, causing rebound of pro-oncogenic activity and drug resistance. For example, MEK inhibition leads to PI3K/AKT activation by relieving a negative feedback on ERBB receptors [95]. Also, when B-Raf inhibitors are employed, B-Raf’s downstream cancerous activity is inhibited as desired; but the inhibitors simultaneously block B-Raf’s suppressive activity on upstream components (the negative feedback effects), causing re-activation of EGFR and Ras [30, 37, 92, 96, 97]. Whether Erks’ inhibitors may have similar effects on upstream components has not been reported so far.

The linkage between suppression of Erk1^R84H^ phosphorylation/activity and the cancerous phenotype is not trivial to explain. It could be that cells in which Erk1^R84H^ activity is very low are selected, as higher Erk’s activity causes senescence and/or cell death. Namely, the founders of tumors (or *foci* in culture) are actually cells that survived the presence of an oncogene by reducing its activity. This notion is supported by observations that induction of oncogenic activity in already transformed cells results in cell death [98]. It is also supported by an experiment of Gillies et al., in which expression of different Ras mutants were induced in culture cells, resulting in various levels of Erk activity. Not all cells in this model were oncogenically transformed, and those that were displayed certain, not necessarily very high levels of Erk [56]. Our results argue that very low Erk1^R84H^’s activity, below our detection abilities, suffices for maintaining the morbidity. A somewhat paradoxical and counterintuitive implication of these findings is that a beneficial therapeutic strategy in some cancers might be activation, rather than inhibition of Erk.

Indeed, the effect of Erk inhibitors on the pathway is reminiscence of that caused by B-Raf inhibitors, namely re-activation of the suppressed cascades. In the case of B-Raf inhibitors this effect promoted disease recurrence and changes in treatment protocols [30, 37, 92, 96, 97]. Perhaps applying Erk inhibitors in the clinic will similarly cause rapid recurrence, so that providing them together with inhibitors of upstream components, such as MEK1/2, as is the current practice for B-Raf inhibitors, would be beneficial.

## Supplementary information

Supplemental data

Supplemental file S3

Supplemental file S4

## Data Availability

The full proteome and phosphoproteome datasets generated in this study have been deposited in the ProteomeXchange Consortium via the PRIDE partner repository with the dataset identifier PXD058196 for the global proteomics and PXD056168 for the phosphoproteome.
