# Supplementary material for: Erk1R84H is an oncoprotein that causes hepatocellular carcinoma in mice and imposes a rigorous negative feedback loop
Source: Oncogene. 2025 May 20;44(31):2689–714. doi: 10.1038/s41388-025-03437-6 (PMC12301236; doi:10.1038/s41388-025-03437-6)
Supplement: Supplementary file 1 — Supplemental data [file 41388_2025_3437_MOESM1_ESM.docx]

**Supplementary Data**

**Supplementary file S1:**

**Establishment of the hepatic-Erk1^R84H^ transgenic mouse model**

The carrier mouse system was constructed to harbor two expression cassettes, integrated into the Rosa26 locus (Fig. 1A). One cassette contains the gene encoding the reverse tetracycline-controlled transcription factor (rtTA) positioned downstream of a strong promoter. But, this promoter remains transcriptionally inactive due to the presence of an intervening sequence, flanked by loxP sites (marked ‘stop’ in Fig. 1A). The other cassette contains the cDNA encoding N-terminally flag-tagged-Erk1^R84H^, positioned under the control of a promoter regulated by rtTA (Fig. 1A).

To achieve tissue-specific expression of Erk1^R84H^, the carrier mouse should be crossed with a mouse expressing Cre-recombinase in the desired tissue (Cre driver mouse). Here we employed an Alb-Cre driver mouse, in which the Cre recombinase is driven by the liver-specific promoter of the albumin gene, resulting in Cre expression specifically in the liver. Through this crossbreeding, heterozygous progeny carrying both the Erk1^R84H^ and Alb-Cre transgenes (heterozygous for the two genes) were obtained, termed hepatic-hetero-Erk1^R84H^ mice. The mice genotypes were confirmed through PCR analysis (Fig. 1), and they were further inbred to obtain mice carrying two copies of the Erk1^R84H^ allele, termed hepatic-homo-Erk1^R84H^ mice. The latter were also validated through PCR analysis (Fig. 1).
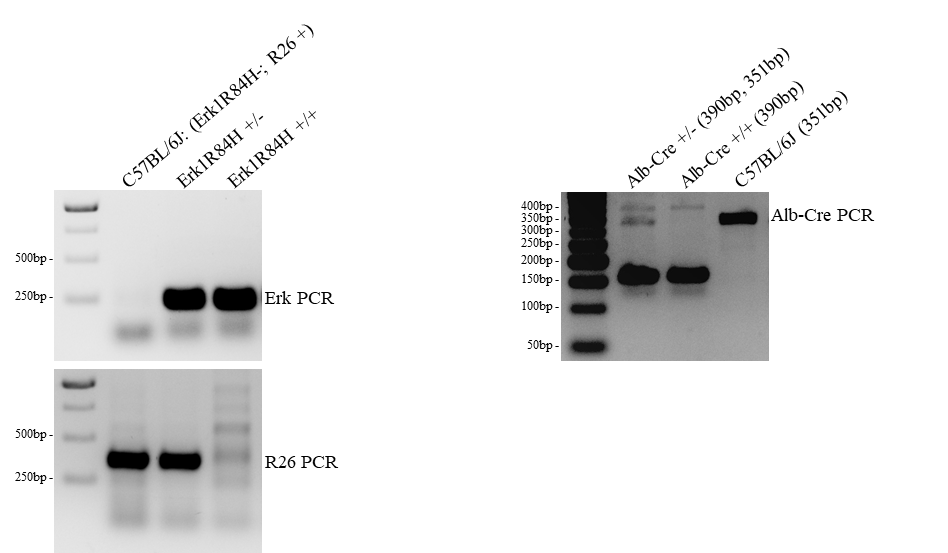


In both the hepatic-hetero-Erk1^R84H^ and hepatic-homo-Erk1^R84H^ mice the intervening sequence between the rtTA gene and its promoter is expected to be excised by the Cre recombinase specifically in the liver, resulting in the expression of functional rtTA. However, rtTA functions as a transcriptional activator only following its binding to a tetracycline or doxycycline (dox) molecule. Consequently, the inducible expression of Erk1^R84H^ in the liver can be achieved by providing dox in the diet.

Figure 1. A representative PCR of genotyping the progeny of inbreeding of Erk1^R84H^ and Alb-cre mice

**Supplementary file S2:**

**The intrinsically active, oncogenic variants of Erk1/2 maintain sensitivity to Erks’ pharmacological inhibitors**

Compounds targeting Erk1/2 were so far assayed only against native Erk molecules, leaving it unknown whether Erk1^R84H^ and Erk1^R84S^ are sensitive or resistant to them *(32, 103, 104)*. This question is important beyond the desire to use the inhibitors in this study, as it could be clinically important given that Erk1^R84H^ appears in human tumors. We thus exposed purified recombinant Erk1^R84H^ and Erk1^R84S^ proteins, as well as MEK-phosphorylated Erk1^WT^, to 3 Erk1/2-specific pharmacological inhibitors, SCH772984, BVD-523 and GDC-0994 *(22, 29, 52, 53)*, in an *in vitro* kinase assays with [γ^32^-P] ATP and myelin basic protein (MBP) as substrates. It was found that all three inhibitors effectively suppressed the activity of purified Erk1^WT^ (MEK-activated), Erk1^R65S^, and Erk1^R65H^ proteins in a similar manner (Fig. 2A). We also tested drug-sensitivity of intrinsically active Erk2^WT^ and mutants and obtained similar results (Fig. 2B).

We further tested whether SCH772984, BVD-523 and GDC-0994 also affect autophosphorylation, as this reaction acts via a different mechanism than that of substrate phosphorylation *(105)*. We incubated purified Erk1^WT^, Erk1^R84S^, and Erk1^R84H^ proteins with the respective inhibitors and radioactive ATP, and collected samples at different time points following reaction initiation (see Materials and Methods). As shown in Fig. 2C, all three compounds inhibited, to various rates, the autophosphorylation capacity of the intrinsically active Erks. GDC-0994 was more potent than the two others.

In summary, SCH772984, BVD-523 and GDC-0994 inhibit both autoactivation and activity of Erk molecules, including of the onco-protein Erk1^R84H^.


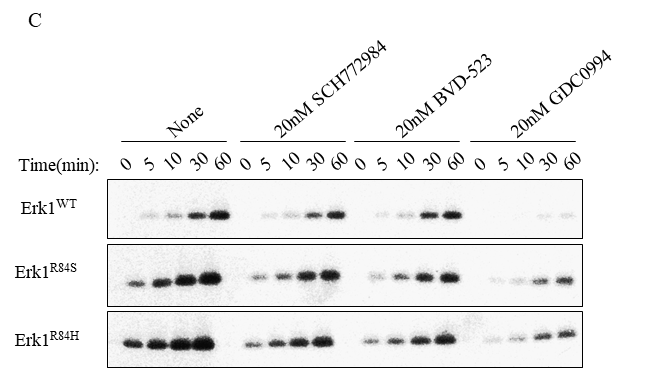

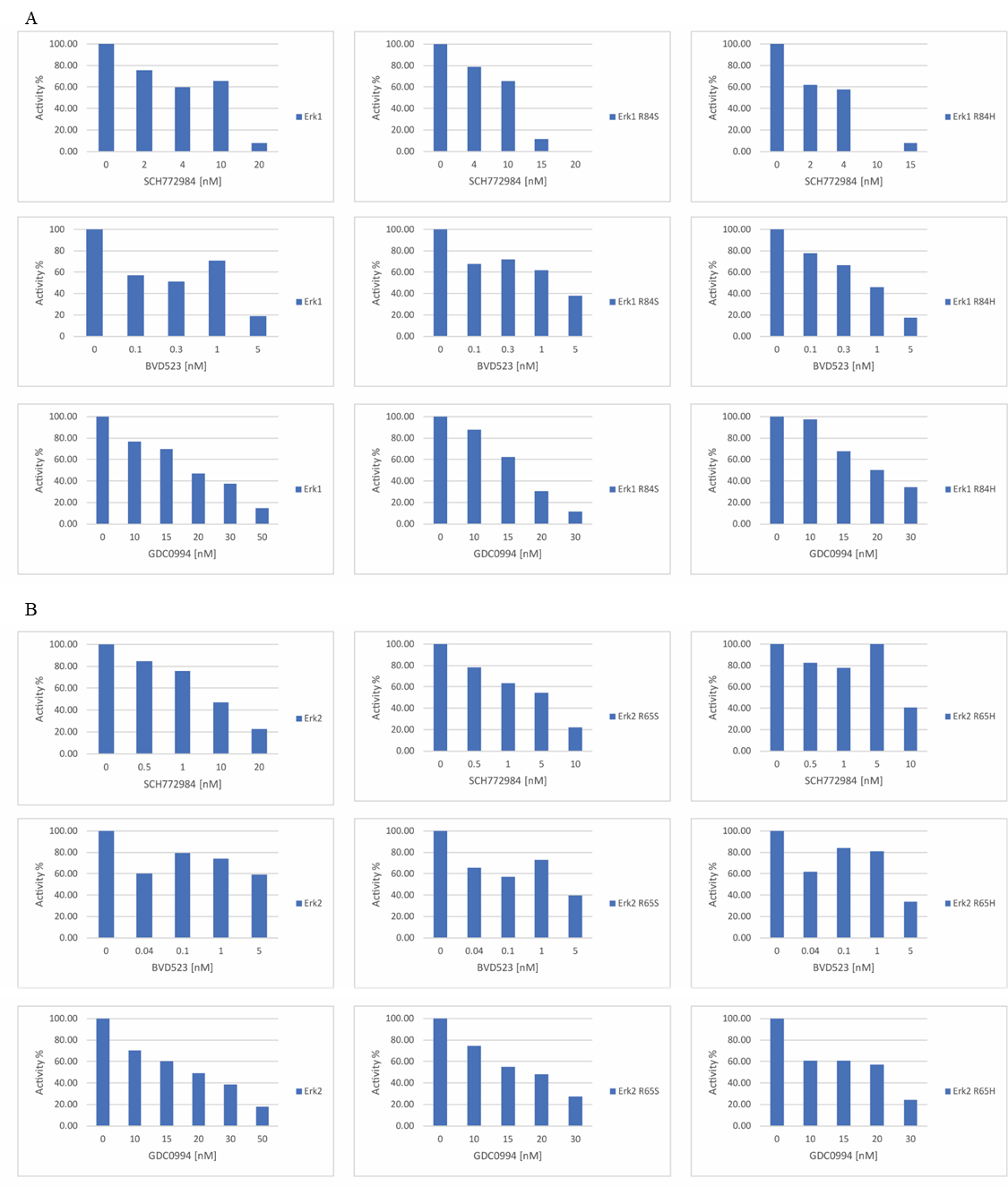


**Figure 2. Erk1^R84S^ and Erk1^R84H^ are sensitive to pharmacological inhibitors of Erk. A.** In vitro kinase assay showing the inhibition of Erk1^WT^ (MEK-phosphorylated), Erk1^R84S^, and Erk1^R84H^ activity by three pharmacological Erk inhibitors: SCH772984, BVD-523, and GDC-0994. All three inhibitors effectively suppressed the kinase activity of both the wild-type and mutant Erk proteins. **B.** Sensitivity of intrinsically active Erk2^WT^ and its mutants (Erk2^R65S^ and Erk2^R65H^) was tested similarly, with comparable inhibitory effects seen across all conditions. **C.** Inhibition of autophosphorylation by SCH772984, BVD-523, and GDC-0994.

**Supplementary file S3:**

This file contains raw data of global expression proteomics data provided as standalone excel sheet

**Table legend:** raw data of global expression proteomics data.

**Supplementary file S4:**

This file contains raw data of phosphoproteomics data provided as standalone excel sheet

**Table legend:** raw data of phosphoproteomics data.

**Supplementary figures**


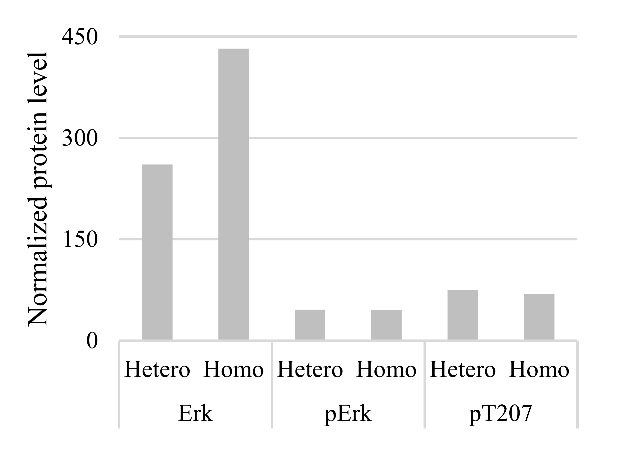


**Fig. S1. Total Erk levels were modestly higher in the hepatic-homo model, while pErk and pT207 levels were comparable between the two models. s**Densitometric analysis of western blots from livers of hepatic-hetero (33 days of expression) and hepatic-homo (35 days of expression) mice. Protein levels were normalized to the average level of GAPDH and plotted as arbitrary units.


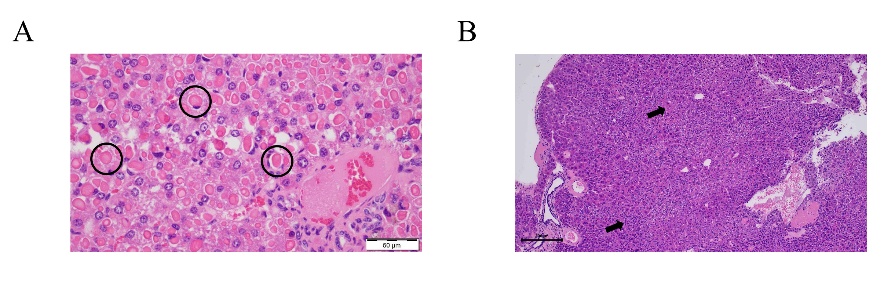


**Fig. S2. Histopathological examination of liver tissues from hepatic-hetero-Erk1^R84H^ mice after 5 months on a dox-supplemented diet.** **A.** H&E staining of cross sections of liver removed from Hepatic-hetero-Erk1^R84H^ showing accumulation of eosinophilic cytoplasmic globules (circled regions) (scale bar, 50 µm). **B.** Cross section of liver removed from Hepatic-hetero-Erk1^R84H^ stained with H&E revealed anisokaryosis (arrows) which is reflected by irregular nuclear size and shape (scale bar = 200 µm).


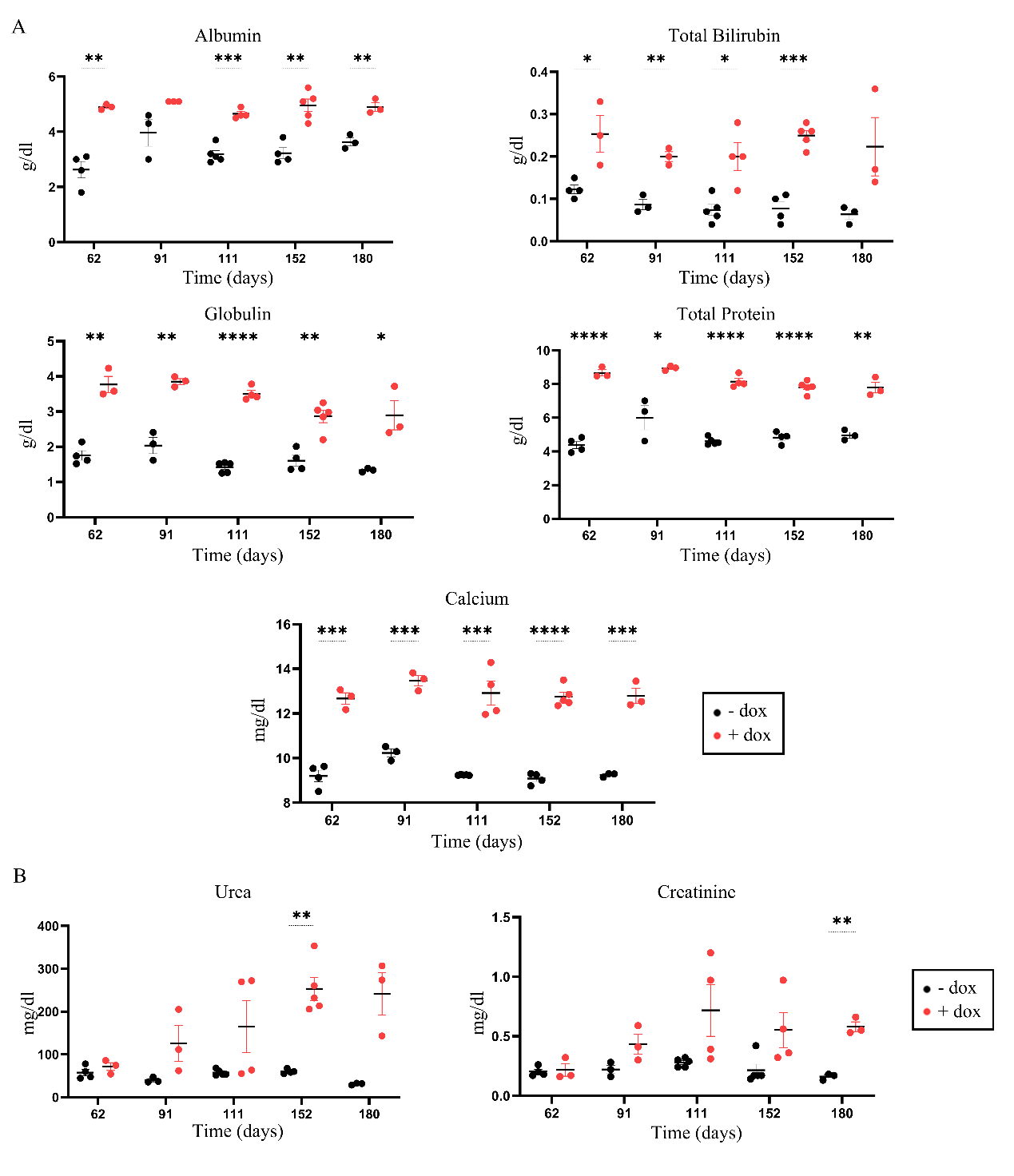


**Fig. S3. Liver function is impaired at early time points in hepatic-homo-Erk1^R84H^ mice, while markers of kidney dysfunction appear later.** Blood serum markers were measured at various time points following induced expressing Erk1^R84H^ in the liver (by provision of dox) and compared to similar mice provided with a regular diet (not expressing Erk1^R84H^. **A.** Liver-associated markers (albumin, total bilirubin, total protein, globulin, and calcium) showed altered values as early as 62 days following induction, indicating early hepatic dysfunction. **B.** Kidney-associated markers (urea and creatinine) remained largely unchanged at early time points following induced expression of Erk1^R84H^, but showed deviations from control values after longer time of Erk1^R84H^ expression.


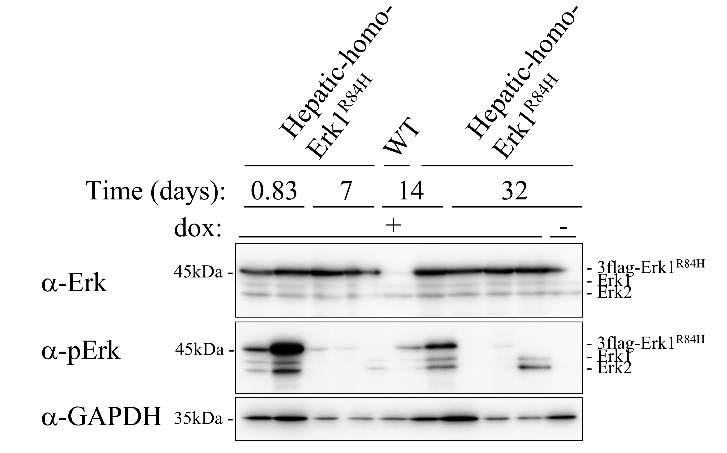


**Fig. S4. Erk1^R84H^ phosphorylation is rapidly downregulated in hepatic-homo-Erk1^R84H^ mice.** Western blot analysis of liver lysates from hepatic-homo-Erk1^R84H^ mice expressing the transgene at the indicated time points following provision of dox-supplemented diet. The levels of total Erk and pErk are shown. Similar to what was observed in hepatic-hetero mice (Fig. 5A), TEY phosphorylation of Erk1^R84H^ decreases rapidly following induction, despite continued expression of the protein.


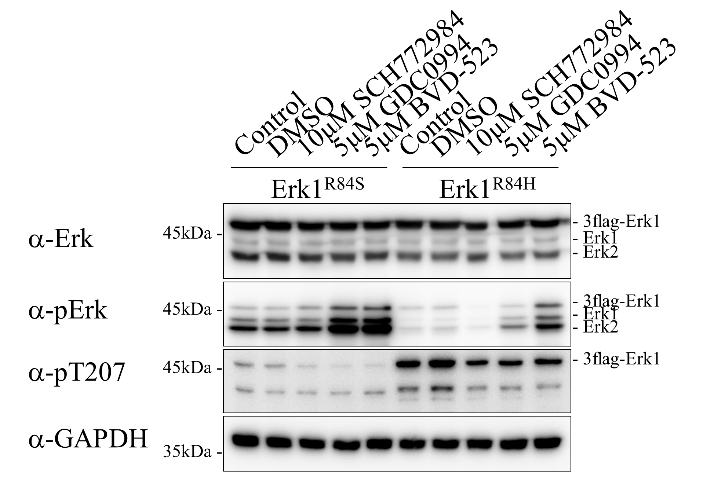


**Fig. S5.** **Effect of different Erk inhibitors on TEY phosphorylation in stable clones expressing Erk1^R84S^ or Erk1^R84H^.** Stable clones were treated with the indicated inhibitors (SCH772984, GDC0994, and BVD-523) for 2 hours at 5 or 10 μM, and protein extracts were subjected to Western blot analysis.
